# Supplementary material for: A modular strategy for extracellular vesicle-mediated CRISPR-Cas9 delivery through aptamer-based loading and UV-activated cargo release
Source: Nat Commun. 2025 Nov 21;16:10309. doi: 10.1038/s41467-025-65995-3 (PMC12639036; doi:10.1038/s41467-025-65995-3)
Supplement: Supplementary file 1 — Supplementary Information [file 41467_2025_65995_MOESM1_ESM.pdf]

A modular strategy for extracellular vesicle-mediated CRISPR-Cas9 delivery through aptamer-based loading and UV-activated cargo release

Omnia M. Elsharkasy*, Charlotte V. Hegeman*, Tom A.P. Driedonks, Xiuming Liang, Ivana Lansweers, Olaf L. Cotugno, Ingmar Y. de Groot, Zoë E.M.N.J. de Wit, Antonio Garcia-Guerra, Niels J.A. Moorman, Sjoerd H. Boonstra, Esmeralda D.C. Bosman, Juliet W. Lefferts, Willemijn S. de Voogt, Jerney J. François, Annet C.W. van Wesel, Samir El Andaloussi, Raymond M. Schiffelers, Sander A.A. Kooijmans, Enrico Mastrobattista, Pieter Vader, Olivier G. de Jong

Supplementary Figures, Tables, and References

Supplementary Figures 1 – 13

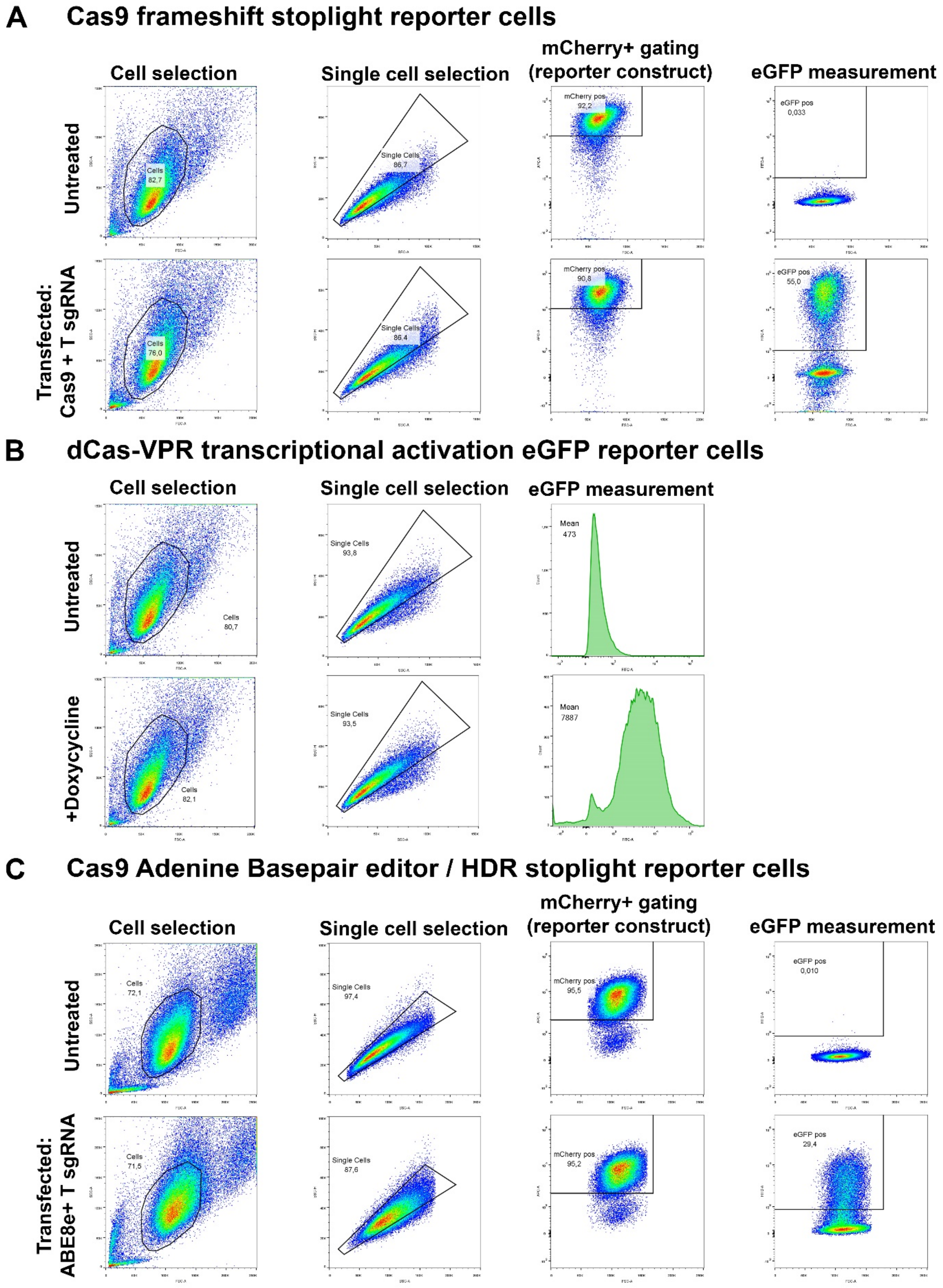

Supplementary Figure 1. Flow cytometry gating strategy employed for the analysis of fluorescent Cas9 activity reporter constructs. a, Gating strategy for analysis of the “stoplight” reporter construct for Cas9 activity-mediated frameshifts (schematic: Fig. 2A). Cells were gated using the forward scatter (FSC-A) and sideward scatter (SSC-A) signals. Single cells were selected using sideward scatter area (SSC-A) and sideward scatter height (SSC-H) signals. Next, mCherry+ cells were selected using FSC-A and mCherry signals to ensure stoplight reporter expression. Lastly, eGFP expression was measured within mCherry+ cells using FSC-A and eGFP signals. Representative plots are shown for untreated HEK293T reporter cells (top row), and HEK293T reporter cells transfected with plasmid DNA for expression of Cas9 and a targeting sgRNA (bottom row). **b,** Gating strategy for analysis of the transcriptional activator reporter construct, based on inducible eGFP expression; pInducer20, dCas9-VPR activity (schematic: Fig. 4A). Cells were gated using the forward scatter (FSC-A) and sideward scatter (SSC-A) signals. Single cells were selected using sideward scatter area (SSC-A) and sideward scatter height (SSC-H) signals. eGFP signal was then measured and mean fluorescence intensity (MFI) was determined. Representative plots are shown for untreated HEK293T reporter cells (top row), and HEK293T reporter cells treated with doxycycline (positive control, bottom row). **c,** Gating strategy for analysis of the “stoplight” reporter construct for adenine base editor (ABE) activity (schematic: Fig. 5A). Cells were gated using the forward scatter (FSC-A) and sideward scatter (SSC-A) signals. Single cells were selected using sideward scatter area (SSC-A) and sideward scatter height (SSC-H) signals. Next, mCherry+ cells were selected using FSC-A and mCherry signals to ensure stoplight reporter expression. Lastly, eGFP expression was measured within mCherry+ cells using FSC-A and eGFP signals. Representative plots are shown for untreated HEK293T reporter cells (top row), and HEK293T reporter cells transfected with plasmid DNA for expression of ABE8e and a T sgRNA (bottom row).

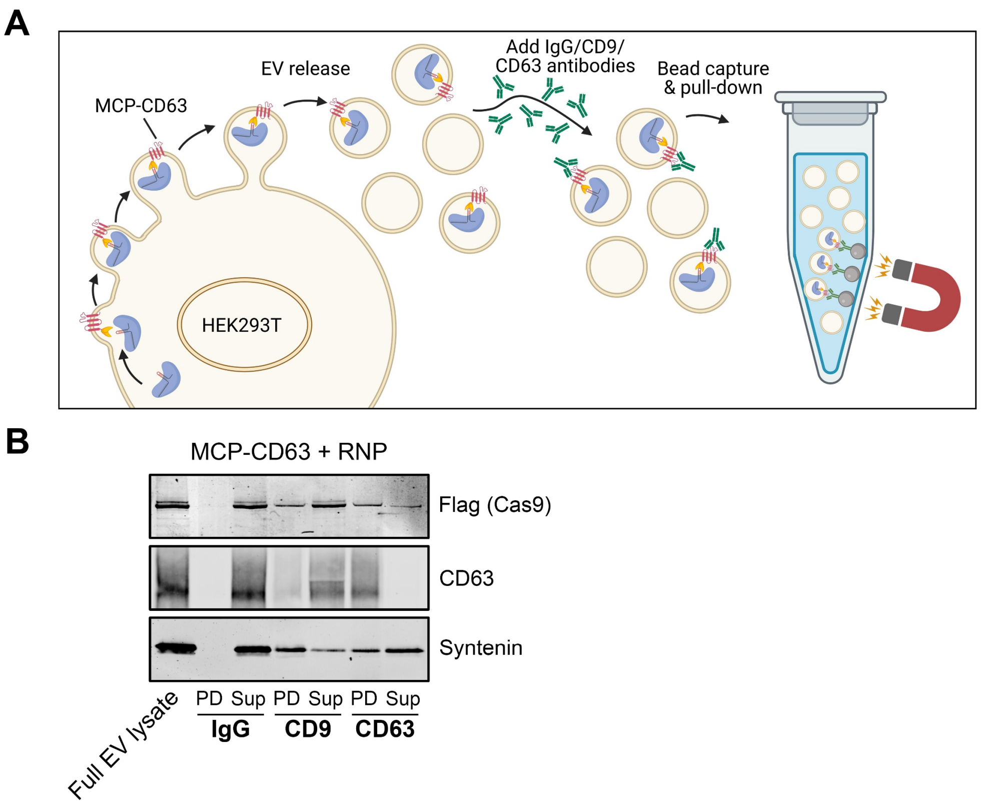

Supplementary Figure 2. Antibody-mediated pulldown of MCP-CD63 RNP-loaded EVs. a, Schematic of the experimental approach for MCP-CD63 RNP-loaded EV capture. EVs are loaded with Cas9 RNPs using MCP-CD63, incubated with either CD9 or CD63-targeting antibodies or an isotype IgG control, washed, and subsequentially captured using magnetic Protein G-coated beads. After capture, supernatant (Sup) or pulled down (PD) samples are lysed and analyzed through western blot analysis. Created in BioRender. Utrecht University, P. (2025) https://BioRender.com/i9mpinj. **b,** Western blot analysis of antibody-captured MCP-CD63-RNP EVs. Syntenin is included as an additional EV marker. Only CD63 antibody-mediated pulldown shows an increase in Cas9 (Flag) signal as compared to its respective supernatant sample. CD9 capture also results in some CD63 and Cas9 pulldown, but the majority of both proteins remains in the supernatant. Non-targeting IgG-mediated capture does not result in capture of Cas9 or either of the EV markers.

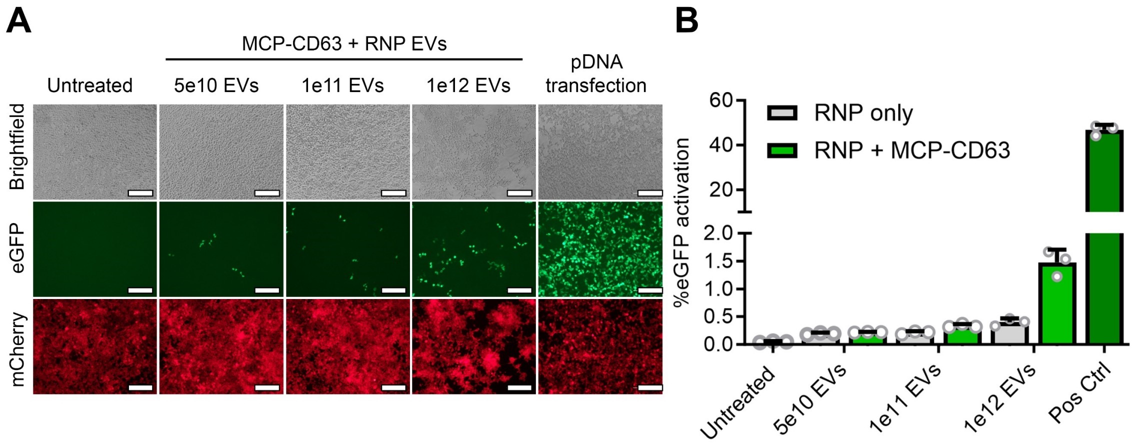

Supplementary Figure 3. Dose response of MCP-CD63 EV RNP delivery. a, b, Fluorescence microscopy **(a)** and flow cytometry analysis **(b)** of HEK293T cells expressing the stoplight reporter construct (Fig. 2A), 72 hours after addition of EVs isolated from HEK293T cells expressing Cas9 + MS2-sgRNA + VSV-G (RNP only) or expressing Cas9 + MS2-sgRNA + MCP-CD63 + VSV-G (RNP + MCP-CD63). Scalebar represents 200 µm. Means + SD, n = 3 biologically independent samples.

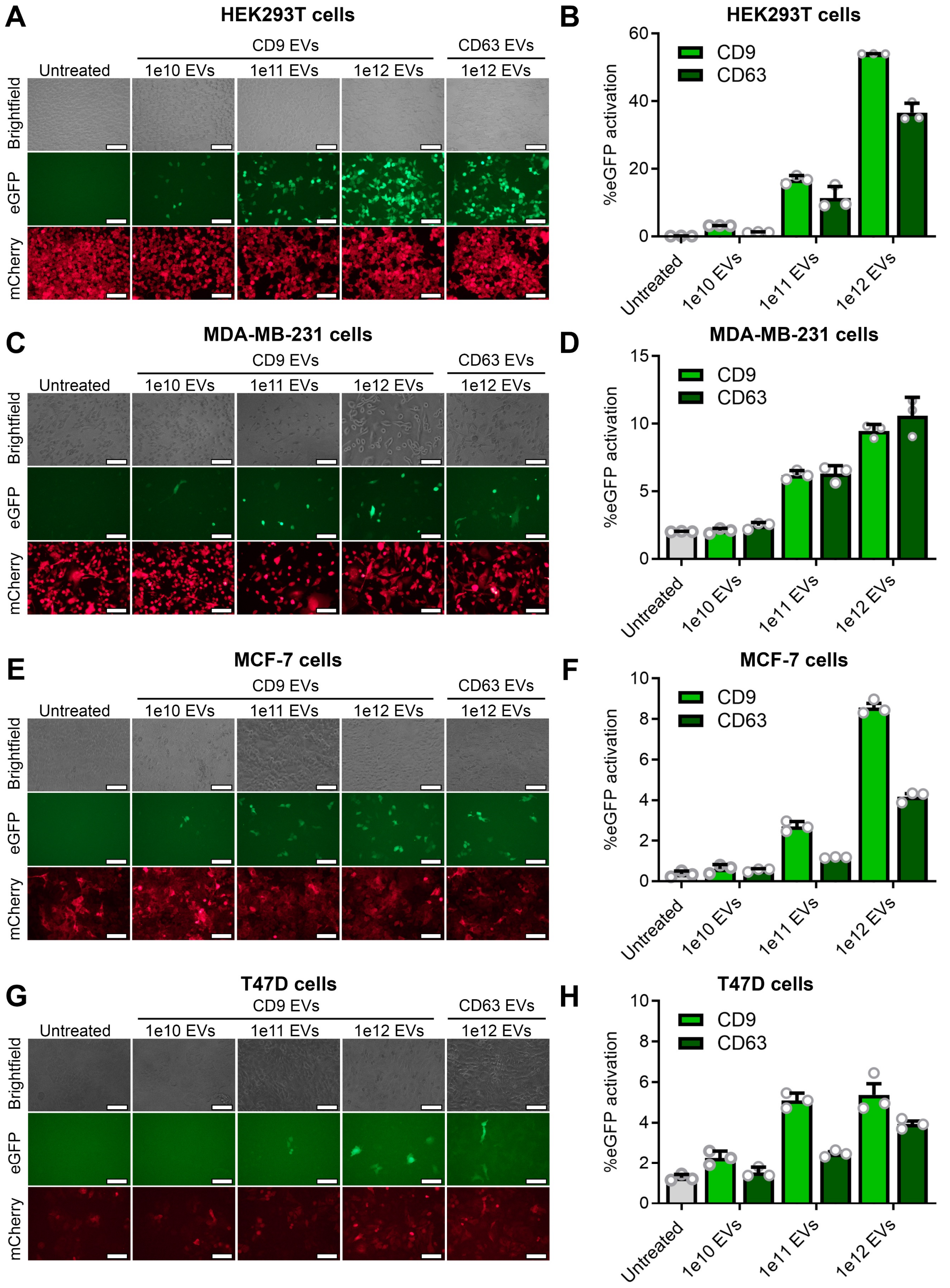

Supplementary Fig 4. Comparison of MCP-PhoCl-CD63 EV-mediated Cas9 delivery to various reporter cell lines. Fluorescence microscopy (left) and flow cytometry analysis (right) of MCP-PhoCl-CD63 VSV-G^{+} EV-mediated Cas9 delivery to HEK293T stoplight cells (**a, b**), MDA-MB-231 stoplight cells (**c, d**), MCF-7 stoplight cells (**e, f**), and T47D stoplight cells (**g, h**). Cells were analyzed 72 hours after EV addition at various dosages. Scale bar represents 100 µm. Means ± SD, n =3 biologically independent samples.

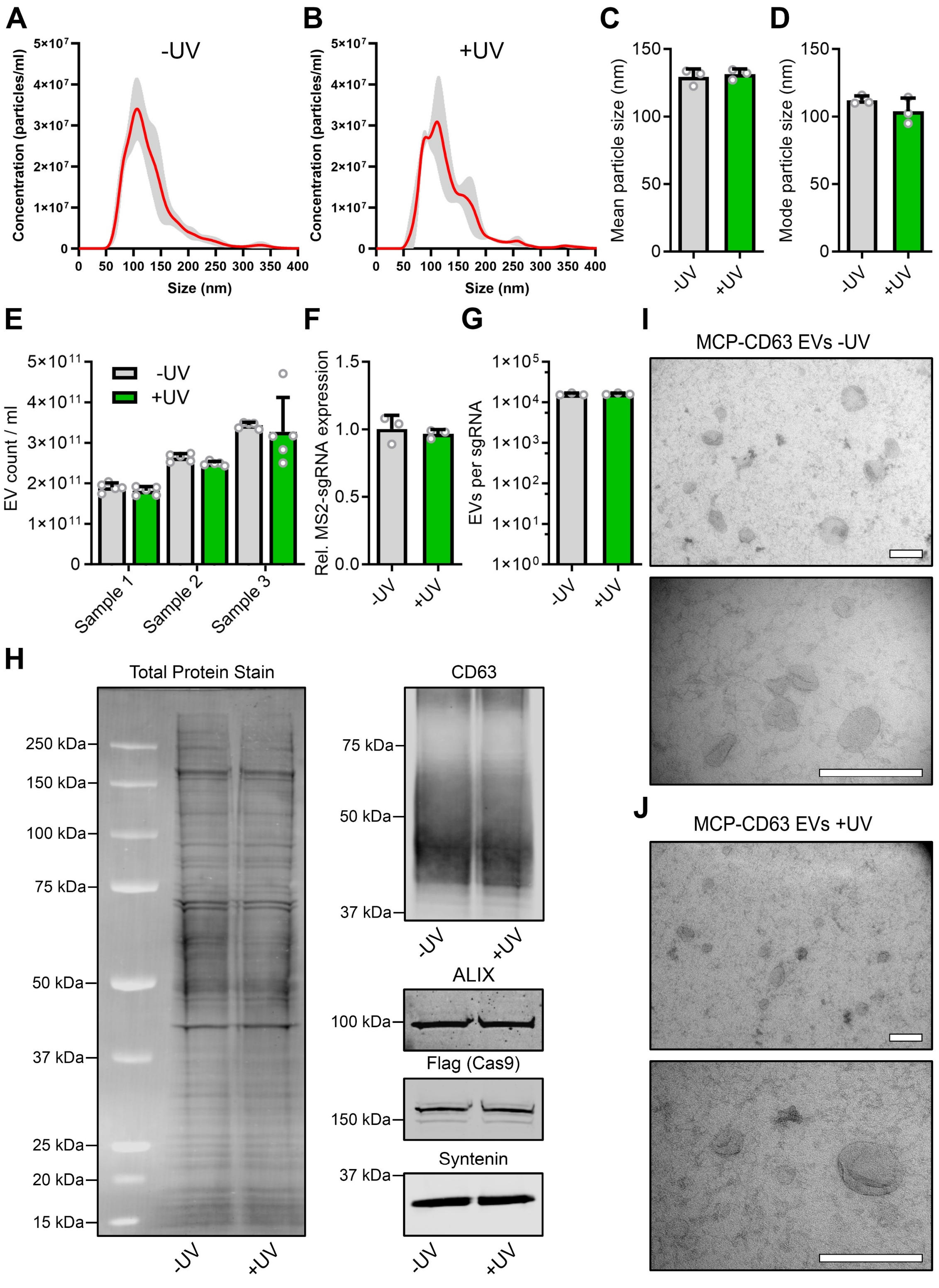

A
-UV
Concentration (particles/ml)
Size (nm)
B
+UV
C
Mean particle size (nm)
D
Mode particle size (nm)
E
EV count / ml
-UV
+UV
Sample 1
Sample 2
Sample 3
F
Rel. MS2-sgRNA expression
G
EVs per sgRNA
I
MCP-CD63 EVs -UV
H
Total Protein Stain
250 kDa
150 kDa
100 kDa
75 kDa
50 kDa
37 kDa
25 kDa
20 kDa
15 kDa
CD63
ALIX
Flag (Cas9)
Syntenin
J
MCP-CD63 EVs +UV

Supplementary Figure 5. Assessment of effects of 395 nm UV-treatment on EV composition. a, b, Nanoparticle Tracking Analysis (NTA) displaying the size distribution of MCP-CD63 RNP-loaded EVs prior to UV treatment **(a)** and after UV treatment **(b)**. Mean $\pm$ SD, n = 3 biologically independent samples. **c, d,** Mean **(c)** and Mode **(d)** particle size of MCP-CD63 RNP-loaded EVs prior to UV treatment (gray) and after UV treatment (green) as measured by NTA analysis. Mean $\pm$ SD, n = 3 biologically independent samples. **e,** Particle count of 3 separate samples of MCP-CD63 RNP-loaded EVs prior to UV treatment (gray) and after UV treatment (green) as measured by NTA analysis. Mean $\pm$ SD, n = 5 technical replicates per biological replicate (n = 3). **f, g** qPCR analysis **(f)** of relative MS2-sgRNA expression in MCP-CD63 RNP-loaded EVs prior to UV treatment (gray) and after UV treatment (green), normalized to a Spike-in RNA, added prior to RNA isolation. Additionally, MS2-sgRNA loading was quantified by ddPCR analysis **(g)**, corrected for NTA particle count and Spike-in RNA, to calculate RNA isolation efficiency, into absolute EV per sgRNA counts Mean $\pm$ SD, n = 3 biologically independent samples. **h,** Western blot analysis of MCP-CD63 RNP-loaded EVs prior to and after UV treatment, showing total protein stain, Cas9 (Flag), and EV markers ALIX, CD63, and syntenin. **i, j,** Representative transmission electron microscopy images of MCP-CD63 RNP-loaded EVs prior to **(i)** and after **(j)** UV treatment, at 2 different levels of magnification. Scalebar represents 200 nm.

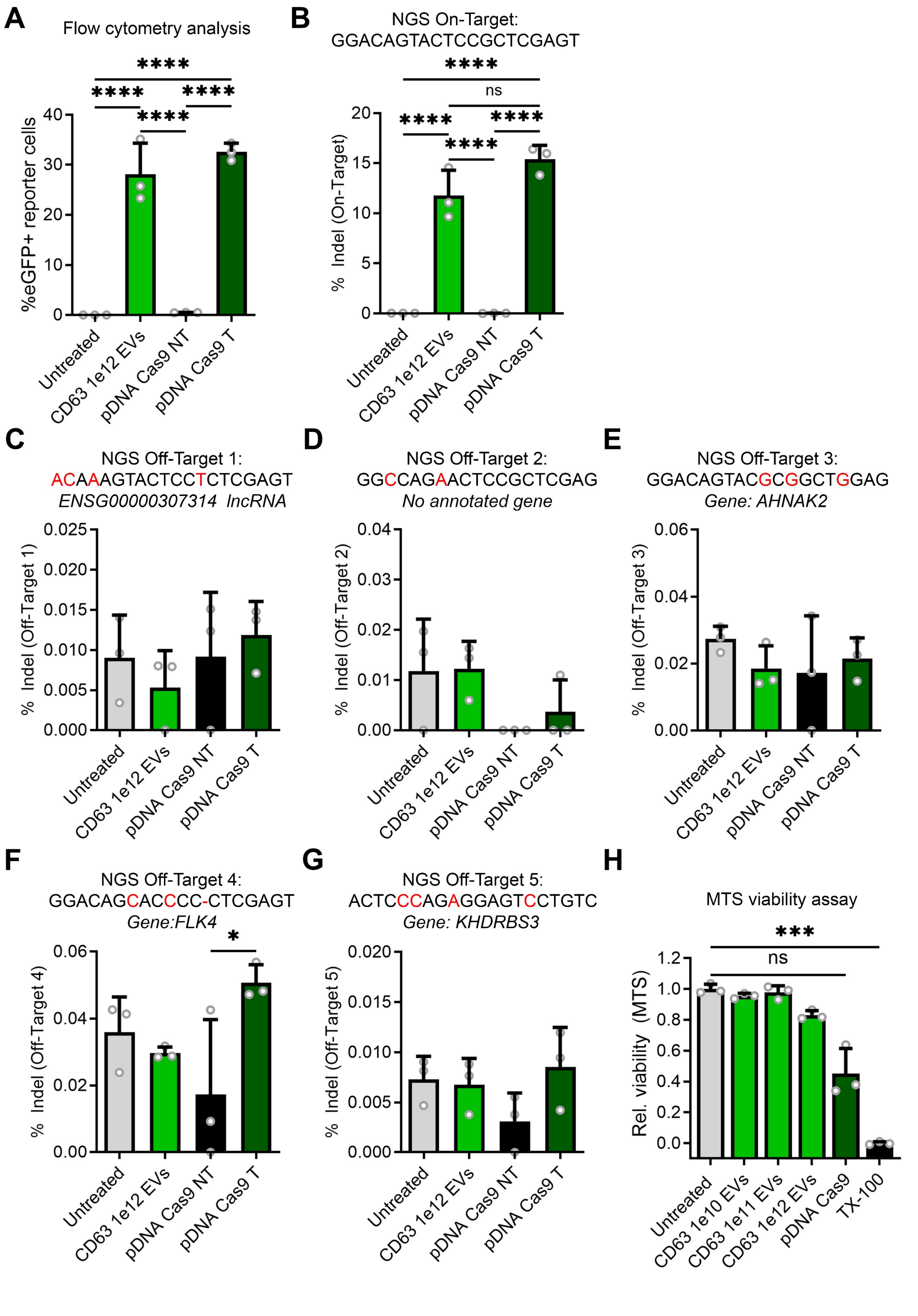

A
Flow cytometry analysis
%eGFP+ reporter cells
B
NGS On-Target:
GGACAGTACTCCGCTCGAGT
% Indel (On-Target)
C
NGS Off-Target 1:
ACAAAGTACTCCTCTCGAGT
ENSG00000307314 lncRNA
% Indel (Off-Target 1)
D
NGS Off-Target 2:
GGCCAGAACTCCGCTCGAG
No annotated gene
% Indel (Off-Target 2)
E
NGS Off-Target 3:
GGACAGTACGCGGCTGGAG
Gene: AHNAK2
% Indel (Off-Target 3)
F
NGS Off-Target 4:
GGACAGCACCCC-CTCGAGT
Gene:FLK4
% Indel (Off-Target 4)
G
NGS Off-Target 5:
ACTCCCAGAGGAGTCCTGTC
Gene: KHDRBS3
% Indel (Off-Target 5)
H
MTS viability assay
Rel. viability (MTS)
Untreated
CD63 1e12 EVs
pDNA Cas9 NT
pDNA Cas9 T
CD63 1e10 EVs
CD63 1e11 EVs
pDNA Cas9
TX-100
ns

Supplementary Figure 6. Off-target activity and cell toxicity of EV-mediated Cas9 delivery and plasmid DNA transfection. a,b HEK293T Stoplight cells were treated with 1.0 x 10^{12} (1e12) MCP-PhoCl-CD63 VSV-G^{+} EVs loaded with Cas9 and a sgRNA targeting the Stoplight reporter construct, or transfected with plasmid DNA (pDNA) encoding for Cas9 and a non-targeting (NT) sgRNA, or a targeting (T) sgRNA, and subsequently analyzed for gene-editing using flow cytometry (**a**) and next generation sequencing (NGS) (**b**). (**c-g**) Furthermore, 5 additional off-target (OT) sites were analyzed by NGS analysis: (**c**) OT-1: chr2:120580702, (**d**) OT-2: chr4:157825214, (**e**) OT-3: chr14:104942109, (**f**) OT-4: chr5:180630776, (**g**) OT-5: chr8:135581932. Means + SD, n = 3 biologically independent samples, One-way ANOVA with Tukey's multiple comparison test. (**h**) A MTS cell viability assay shows no toxicity over the course of 72 hours after various dosages of MCP-PhoCl-CD63 RNP-loaded VSV-G^{+} EVs were added. Plasmid DNA transfection of the same constructs (pDNA Cas9) shows a trend towards a moderate level of toxicity ($p = 0.086$). Toxicity was determined by comparing relative viability to untreated cells. Triton X-100 (1%) treated cells were used as a positive control, which was set to 0% viability. Mean + SD, n = 3 biologically independent samples, One-way ANOVA with Dunnett's multiple comparison test. * = $p < 0.05$, *** = $p < 0.001$.

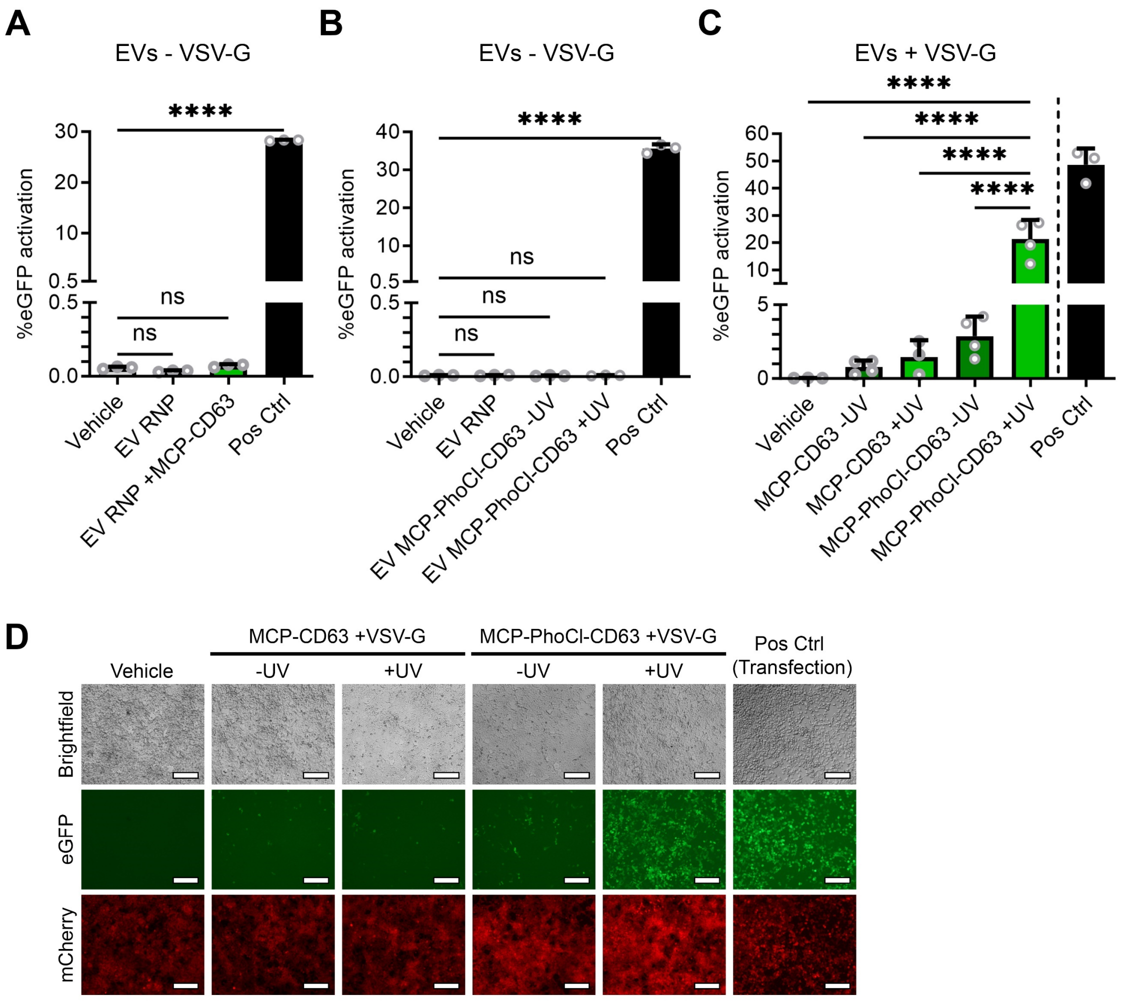

Supplementary Figure 7. Effects of VSV-G and UV on MCP-CD63 and MCP-PhoCl-CD63 Cas9 delivery. a, Flow cytometry analysis of HEK293T cells expressing the stoplight reporter construct for Cas9 activity 72 hours after addition of EVs isolated from HEK293T cells expressing Cas9 + MS2-sgRNA (EV RNP) or expressing Cas9 + MS2-sgRNA + MCP-CD63 (EV RNP + MCP-CD63) without the co-expression of VSV-G shows no detectable EV-mediated functional Cas9 delivery. Means + SD, n = 3 biologically independent samples, One-way ANOVA with Dunnett's multiple comparison test. **b,** Flow cytometry analysis of HEK293T cells expressing the stoplight reporter construct for Cas9 activity 72 hours after addition of EVs isolated from HEK293T cells expressing Cas9 + MS2-sgRNA (EV RNP) or expressing Cas9 + MS2-sgRNA + MCP-PhoCl-CD63 with- (+UV) or without (-UV) UV treatment, without the co-expression of VSV-G shows no detectable EV-mediated functional Cas9 delivery. Means + SD, n = 3 biologically independent samples, One-way ANOVA with Dunnett's multiple comparison test. **c, d** Flow cytometry analysis **(c)** and fluorescence microscopy images **(d)** of HEK293T cells expressing the stoplight reporter construct for Cas9 activity 72 hours after addition of EVs isolated HEK293T cells expressing VSV-G, Cas9 and a targeting MS2-sgRNA alongside MCP-CD63 or MCP-PhoCl-CD63, either treated with- or without UV. MCP-PhoCl-CD63 with UV significantly outperforms all other conditions. UV treatment does not result in decreased Cas9 delivery in MCP-CD63 EVs, indicating that cargo is not negatively affected by UV. Means + SD, n = 3 biologically independent samples, One-way ANOVA with Tukey's multiple comparison test (plasmid DNA transfection "Pos Ctrl" excluded from multiple comparison's test). 5.0 x 10^{11} EVs were added per well. Scalebar represents 200 µm. **** *p*< 0.0001.

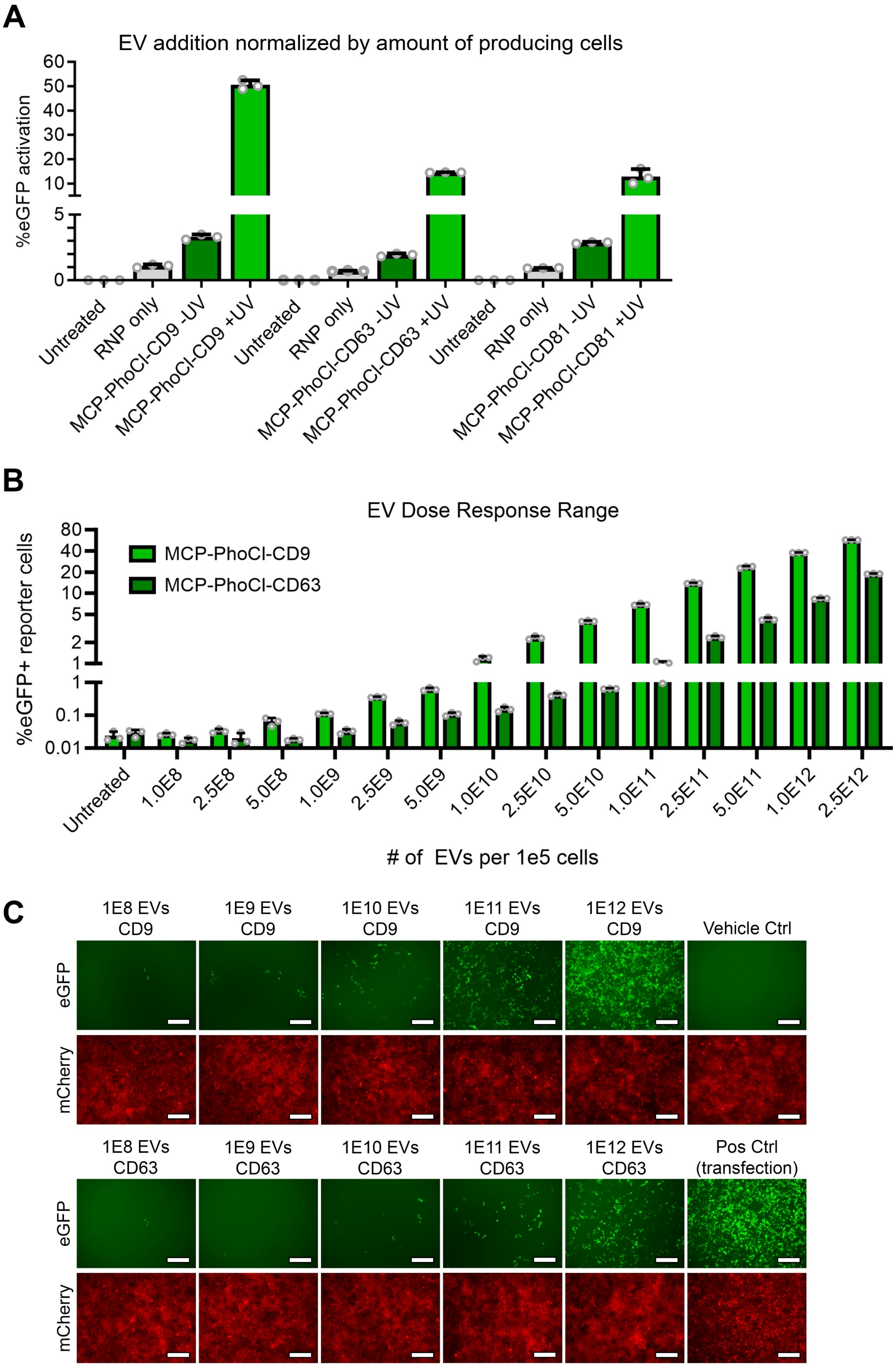
A
EV addition normalized by amount of producing cells
%eGFP activation
60
50
40
30
20
10
5
0
Untreated
RNP only
MCP-PhoCl-CD9 -UV
MCP-PhoCl-CD9 +UV
Untreated
RNP only
MCP-PhoCl-CD63 -UV
MCP-PhoCl-CD63 +UV
Untreated
RNP only
MCP-PhoCl-CD81 -UV
MCP-PhoCl-CD81 +UV
B
EV Dose Response Range
%eGFP+ reporter cells
80
40
20
10
5
2
1
1
0.1
0.01
MCP-PhoCl-CD9
MCP-PhoCl-CD63
Untreated
1.0E8
2.5E8
5.0E8
1.0E9
2.5E9
5.0E9
1.0E10
2.5E10
5.0E10
1.0E11
2.5E11
5.0E11
1.0E12
2.5E12
# of EVs per 1e5 cells
C
1E8 EVs CD9
1E9 EVs CD9
1E10 EVs CD9
1E11 EVs CD9
1E12 EVs CD9
Vehicle Ctrl
eGFP
mCherry
1E8 EVs CD63
1E9 EVs CD63
1E10 EVs CD63
1E11 EVs CD63
1E12 EVs CD63
Pos Ctrl (transfection)
eGFP
mCherry

Supplementary Figure 8. Comparison of tetraspanins in MCP-PhoCl constructs normalized on cell count and particle count. a, Comparison of MCP-CD9-PhoCl, MCP-CD63-PhoCl, and MCP-CD81-PhoCl normalized on cell count of EV-producing cells co-expressing VSV-G, Cas9 and MS2-sgRNA. EVs from 5.0 x10^8 cells were added per well. Relative differences in Cas9 delivery efficiency between CD9, CD63 and CD81 is comparable to experiments normalized to particle count (shown in Fig. 3). Means + SD, n = 3 biologically independent samples. **b, c** Flow cytometry analysis **(b)** and fluorescence microscopy images **(c)** of HEK293T cells expressing the stoplight reporter construct for Cas9 activity 72 hours after addition of an EV dose-range, between 1.0 x 10^8 and 2.5 x 10^{12} EVs per well, from HEK293T cells expressing VSV-G, Cas9 and a targeting MS2-sgRNA alongside MCP-PhoCl-CD9 and MCP-PhoCl-CD63, confirming a dose-dependent delivery of Cas9. Scale bar represents 200 µm. Means + SD, n = 3 independent experiments.

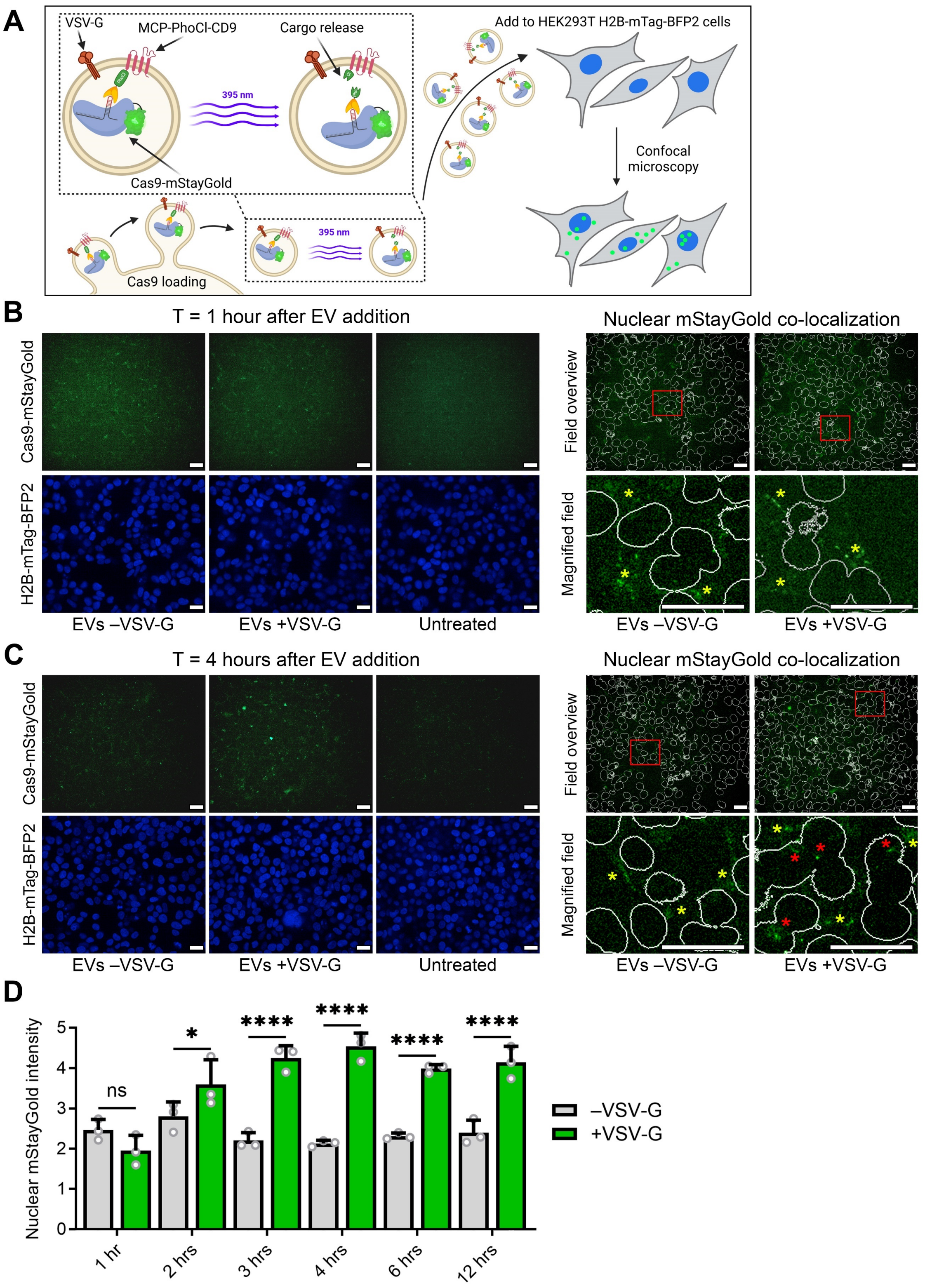
A
VSV-G
MCP-PhoCl-CD9
Cargo release
395 nm
Cas9-mStayGold
Cas9 loading
Add to HEK293T H2B-mTag-BFP2 cells
Confocal microscopy
B
T = 1 hour after EV addition
Cas9-mStayGold
H2B-mTag-BFP2
EVs –VSV-G
EVs +VSV-G
Untreated
Nuclear mStayGold co-localization
Field overview
Magnified field
C
T = 4 hours after EV addition
D
Nuclear mStayGold intensity
ns
*
****
1 hr
2 hrs
3 hrs
4 hrs
6 hrs
12 hrs
–VSV-G
+VSV-G

Supplementary Figure 9. Co-expression of VSV-G facilitates EV-mediated Cas9 delivery. a, Schematic of the experimental approach to study the effect of co-expression of VSV-G on EV-mediated Cas9 delivery. Fluorescently tagged Cas9 with the highly bright green fluorescent protein mStayGold[1] was co-expressed with MS2 sgRNAs and loaded into EVs using the MCP-PhoCl-CD9 construct, either with- or without VSV-G co-expression. EVs were isolated, treated with UV, and added to HEK293T cells stably expressing mTag-BFP2 Histone 2B (H2B-mTag-BFP2), as fluorescently tagged H2B expression gives high levels of nuclear fluorescence[1], constitutively presenting a high blue fluorescent signal in their nuclei. Cells were imaged up to 12 hours after EV addition using a live-imaging confocal system. Created in BioRender. Utrecht University, P. (2025) https://BioRender.com/s73l75q. **b, c,** Live-imaging confocal microscopy images of H2B-mTag-BFP2 HEK293T cells, 1 hour **(b)** and 4 hours **(c)** after EV addition, at 60x magnification. mStayGold-Cas9 (top left) and H2B-mTag-BFP2 (bottom left) are imaged on untreated cells, and on cells treated with mStayGold-Cas9 RNP-loaded EVs with- or without co-expression of VSV-G. Blue fluorescent signal is then used to determine surface areas of the nucleus (top right). Increased magnification (bottom right) shows mStayGold-Cas9 localization in relation to nuclei. After 1 hour, the majority of green fluorescent signal is present outside of the nuclei, as indicated by a yellow asterisk (*). At later timepoints green fluorescent signal is also observed inside the nuclei, as is indicated by a red asterisk. Scalebar represents 25 µm. **d,** Quantification of mean green fluorescent signal in nuclei over time. Average green fluorescent intensity was measured in 4 imaging fields at 60x magnification (as shown in the left of panel **b** and **c**) for 3 biological replicates, over the course of 12 hours. Fluorescence intensity was corrected for average intensity signal of untreated cells. To ensure equal levels of Cas9-mStayGold addition, EV particle count was normalized to the fluorescent signal of 1.5×10^{11} VSV-G+ EVs. Mean $\pm$ SD, n = 3 biologically independent samples, Sidak's multiple comparison's test. * = $p < 0.05$, **** = $p < 0.0001$.

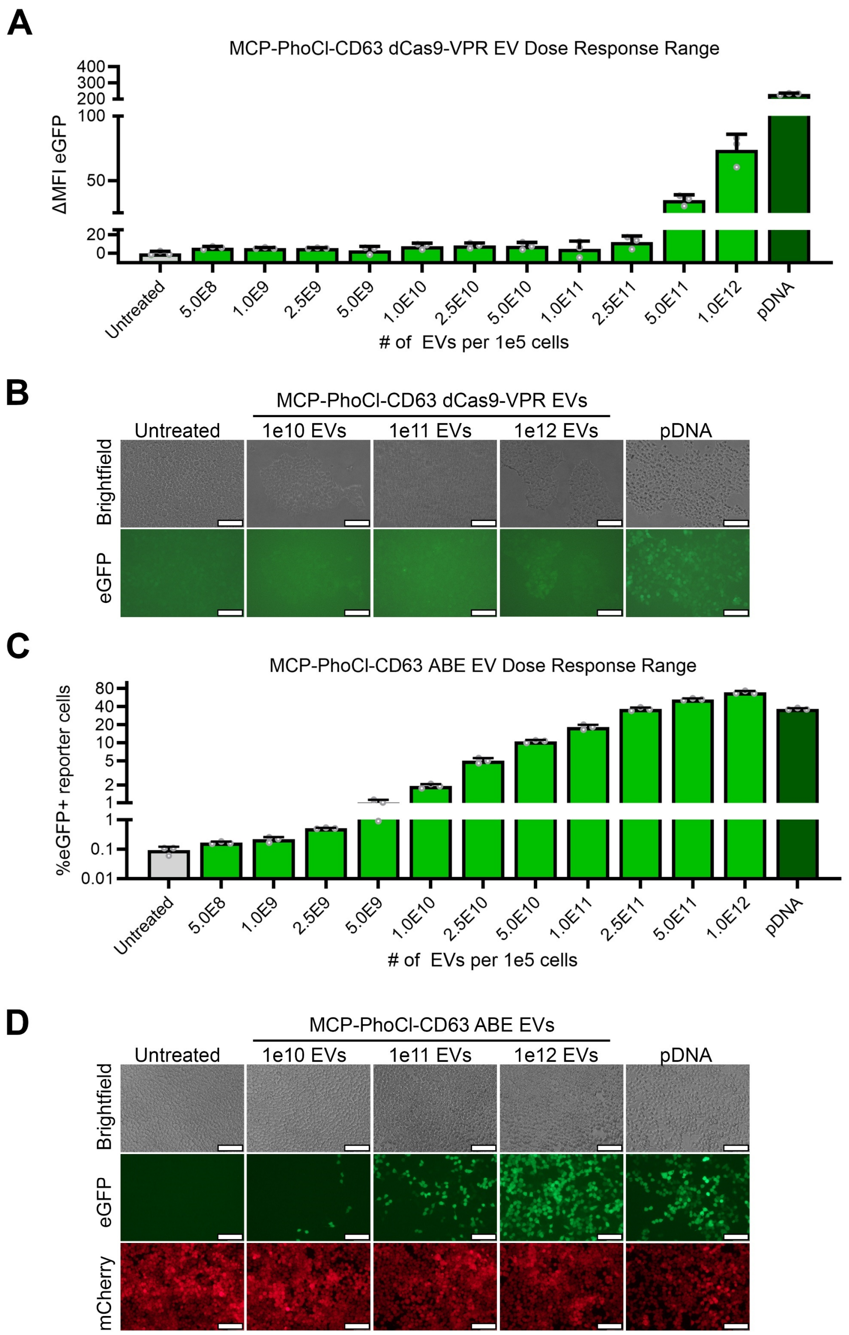

Supplementary Fig 10. Dose response of MCP-PhoCl-CD63 EV-mediated delivery of dCas9-VPR and ABE8e. a, b Flow cytometry analysis (**a**) and fluorescence microscopy (**b**) of HEK293T cells expressing the inducible eGFP reporter construct for transcriptional activation, 48 hours after addition of an EV dose-range, between 5.0 x 10^8 and 1.0 x 10^{12} EVs per well, delivering dCas9-VPR. Means + SD, n = 3 independent experiments. **c, d** Flow cytometry analysis (**c**) and fluorescence microscopy (**d**) of HEK293T cells expressing the ABE stoplight reporter construct, 72 hours after an addition of an EV dose range, between 5.0 x 10^8 and 1.0 x 10^{12} EVs per well, delivering ABE8e. Both experiments include a positive control consisting of pDNA transfection expressing dCas9-VPR or ABE8e, respectively (pDNA). Means + SD, n = 3 independent experiments. Scale bar represents 100 µm.

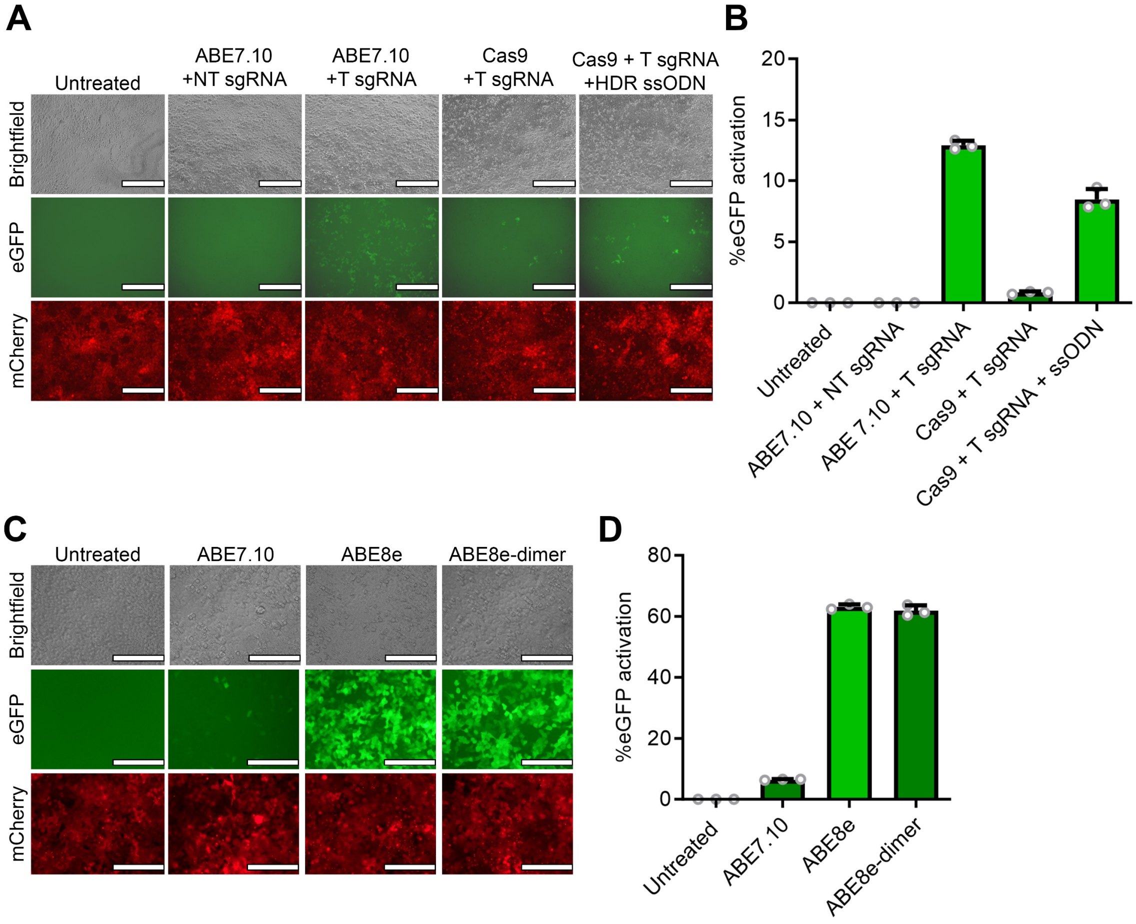

Supplementary Figure 11. Cas9-mediated homology directed repair and adenine base editor comparison on a fluorescent adenine base editor reporter. a,b Fluorescent microscopy images **(a)** and flow cytometry analysis **(b)** of HEK293T cells expressing the stoplight reporter construct for ABE activity, 72 hours after transfection with plasmid DNA expressing ABE7.10 with a non-targeting (NT) or targeting (T) sgRNA, or Cas9 with a T sgRNA with- or without a ssODN HDR template. Transfection of both ABE7.10 and Cas9 with an ssODN template with their T sgRNAs induce eGFP expression. Means + SD, n = 3 biologically independent samples. **c, d** Fluorescent microscopy images **(c)** and flow cytometry analysis **(d)** of HEK293T cells expressing the stoplight reporter construct for ABE activity, 96 hours after transfection with plasmid DNA expressing ABE7.10, ABE8e or ABE8e-dimer with a T sgRNA. ABE8e and ABE8e-dimer show similar activity, and both strongly outperform ABE7.10. Scale bar represents 200 µm. Means + SD, n = 3 biologically independent samples.

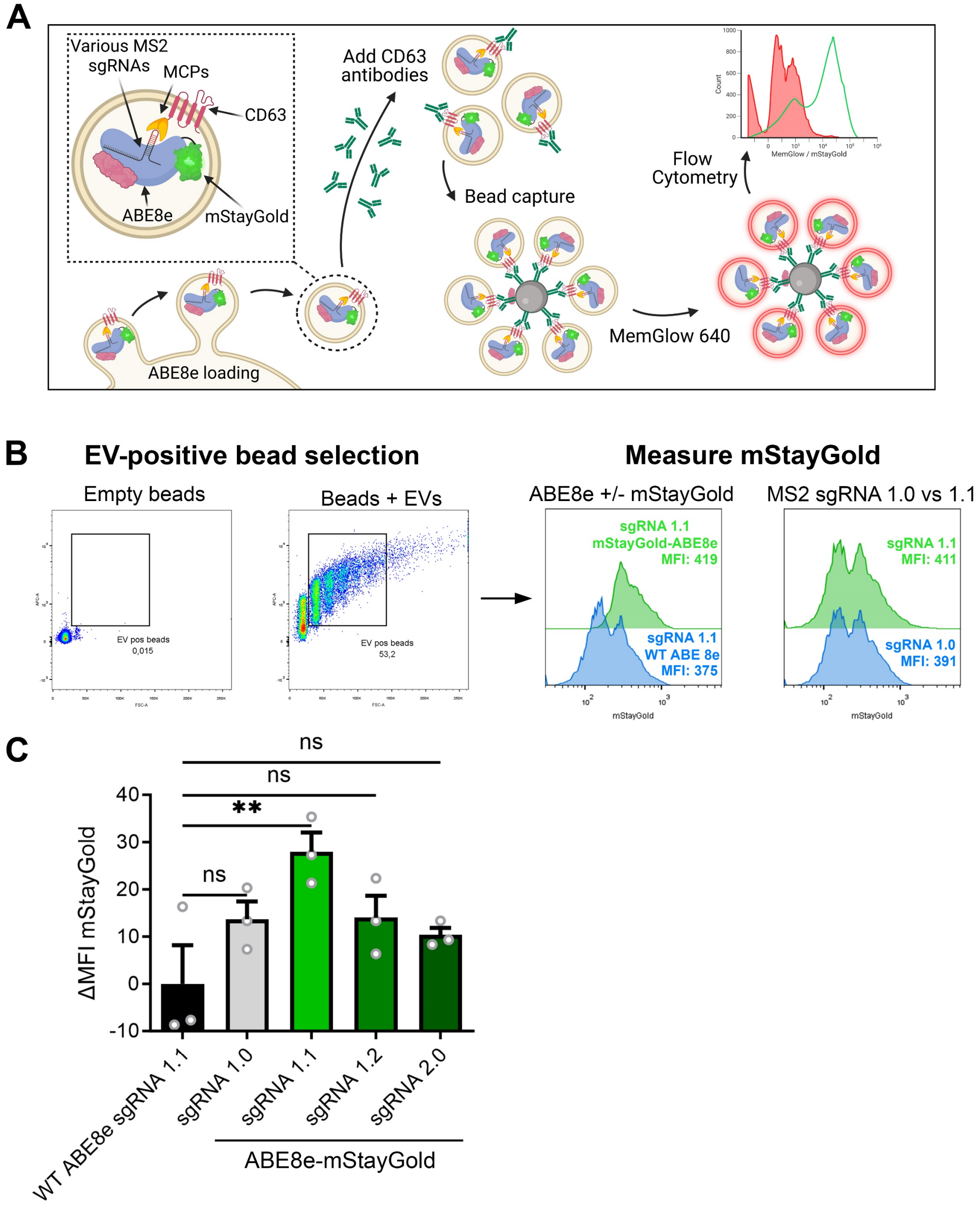

Supplementary Figure 12. MS2 loops in the second stemloop of sgRNA affect ABE8e loading in EVs. a, Schematic of the experimental approach to study ABE8e loading in EVs using different (MS2)-sgRNAs. Fluorescently tagged ABE8e with mStayGold was co-expressed with sgRNAs with MS2 hairpins integrated in the tetraloop (sgRNA 1.1), second stemloop (sgRNA 1.2), both (sgRNA 2.0), or neither (sgRNA 1.0)[2], and loaded into EVs using the MCP-CD63 construct. EVs were isolated, treated with CD63 antibodies, and captured using Protein G-coupled beads. Bead-bound EVs were then stained with MemGlow 640. After washing, EV-loaded beads were then analyzed by flow cytometry to assess green fluorescent signal (ABE8e-mStayGold) and red fluorescent signal (MemGlow 640). Created in BioRender. Utrecht University, P. (2025) https://BioRender.com/u3zui8b. **b,** Gating and analysis strategy. EV-positive beads were gated using FSC-A and APC-A channels. Mean fluorescent intensity was then measured to assess ABE8e-mStaygold loading. Representative green fluorescent histograms are shown for EVs loaded with unlabeled ABE8e vs ABE8e-mStayGold using sgRNA 1.1 (left), and EVs loaded with ABE8e-mStayGold using sgRNA 1.0 vs sgRNA 2.0 (right). **c,** Quantification of mean fluorescent signals on MemGlow 640-positive beads. Fluorescence intensity was corrected for average intensity of beads loaded with unlabeled ABE8e. Only EVs with an MS2 hairpin positioned only in the tetraloop (sgRNA 1.1) showed a significant

increase in ABE8e-mStayGold loading. Mean ± SEM, n = 3 biologically independent samples, One-way ANOVA with Dunnett's multiple comparison test. ** = $p < 0.01$.

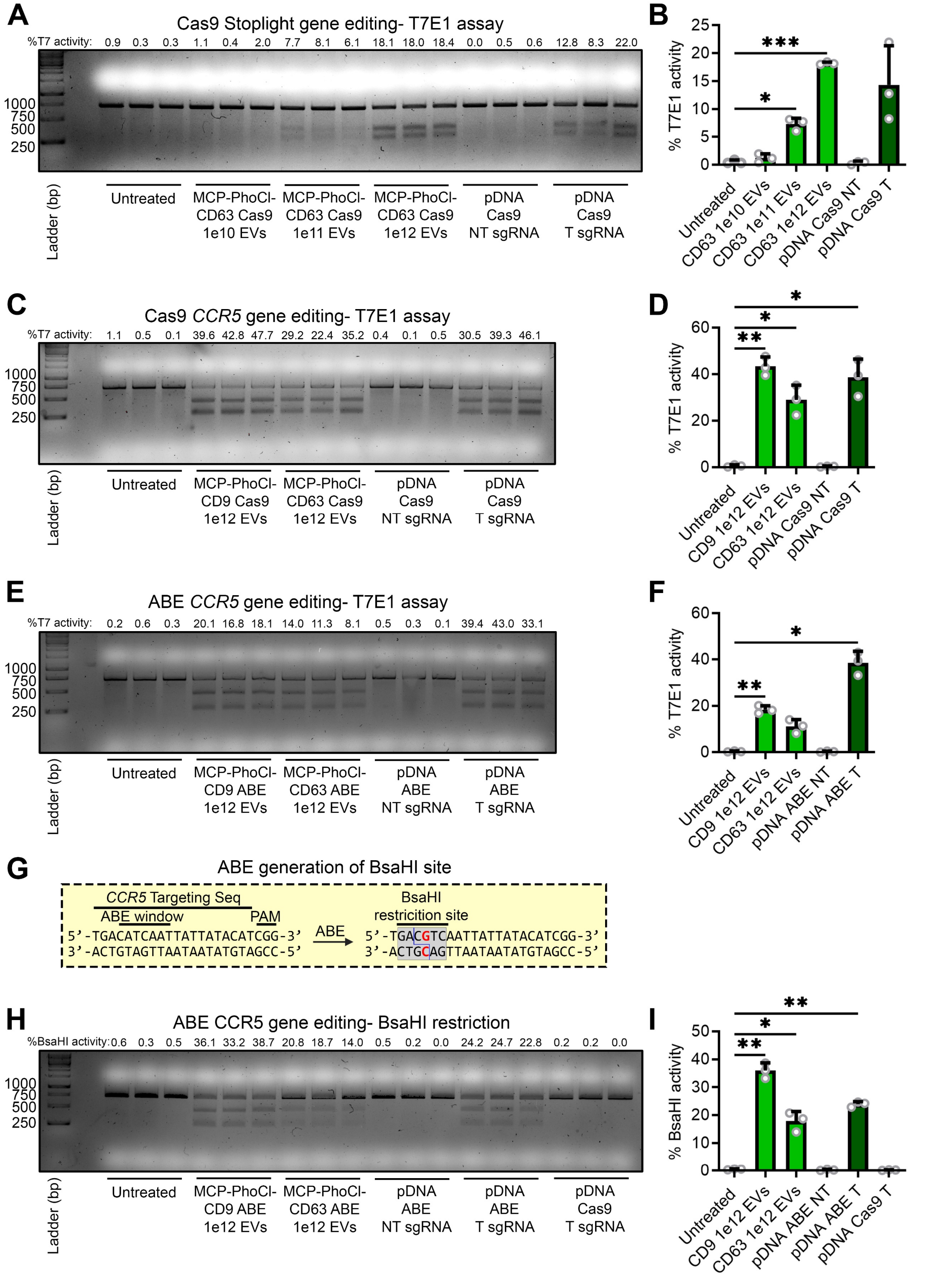

Supplementary Figure 13. Genomic analysis of Stoplight reporter and endogenous *CCR5* gene-editing. a, A T7 endonuclease 1 (T7E1) assay on genomic DNA isolated from HEK293T cells treated with various dosages of MCP-PhoCl-CD63 VSV-G$^+$ EVs, or transfected with plasmid DNA (pDNA) expressing Cas9 with a non-targeting (NT) or targeting (T) sgRNA for the stoplight reporter construct. **b,** Quantification of the T7E1 activity assay on the stoplight reporter locus. **c-e**, A T7E1 assay on genomic DNA isolated from HEK293T cells treated with various dosages of MCP-PhoCl-CD63 and MCP-PhoCl-CD9 VSV-G$^+$ EVs, or pDNA transfection, delivering Cas9 (**c**) or ABE8e (**e**), targeting the *CCR5* locus, including quantification (**d, f**). **g,** ABE8e-mediated base editing of the target *CCR5* sequence produces a BsaHI restriction site, allowing specific enzymatic confirmation of ABE-mediated base editing. **h,i,** BsaHI restriction on the *CCR5* locus after treatment with MCP-PhoCl-CD63 and MCP-PhoCl-CD9 VSV-G$^+$ EVs, or pDNA transfection, delivering ABE8e (**h**) confirms specific ABE-mediated base editing, as quantified in (**i**). Mean + SD, n = 3 biologically independent samples, One-way ANOVA with Dunnett's multiple comparison test. * = $p < 0.05$, ** = $p < 0.01$, *** = $p < 0.001$.

Supplementary Tables 1 – 5

Supplementary Table 1: Ordered oligonucleotide sequences

Target	Purpose	Orientation	Sequence
Cas9 stoplight	sgRNA cloning	Sense	5’-CACCGGGACAGTACTCCGCTCGAGT-3’
Cas9 stoplight	sgRNA cloning	Antisense	5’-AAACACTCGAGCGGAGTACTGTCCC-3
Tet resp. element	sgRNA cloning	Sense	5’-CACCGGTCTCTATCACTGATAGGGAG-3’
Tet resp. element	sgRNA cloning	Antisense	5’-AAACCTCCCTATCAGTGATAGAGACC-3’
ABE stoplight	sgRNA cloning	Sense	5’-CACCGCTTACTTGTACAGCTCGTCC-3’
ABE stoplight	sgRNA cloning	Antisense	5’-AAACGGACGAGCTGTACAAGTAAGC-3’
CCR5 locus	sgRNA cloning	Sense	5’-CACCGTGACATCAATTATTATACAT-3’
CCR5 locus	sgRNA cloning	Antisense	5’-AAACATGTATAATAATTGATGTCAC-3’
ABE Stoplight	HDR template	Antisense	5’-ACGACGCCCGTGAAAAGCTCTTCACCCTTAGACACGGCTT GCTTGTACAGCTCGTCCAAGCCGCCCGTAGAATGCCTGCCT-3’
MS2 sgRNA	ddPCR probe	Sense	5'-/56-FAM/AGGCTAGTC/ZEN/CGTTATCAACTTGGCCAA C/31ABkFQ/ -3'
Spike-in RNA	ddPCR probe	Sense	5'-/SHEX/TCGGCATCC/ZEN/AGACCGTCGGCT/31ABkFQ/ -3’
Adapted from GenBank: KC702164.1 ERCC-00002	Spike-in RNA for RNA isolation efficiency and ddPCR normalization	Sense	5'-rGrCrU rCrCrA rGrArU rUrArC rUrUrC rCrArU rUrUrC rCrGrC rCrCrA rArGrC rUrGrC rUrCrA rCrArG rUrArU rArCrG rGrGrC rGrUrC rGrGrC rArUrC rCrArG rArCrC rGrUrC rGrGrC rUrGrA rUrCrG rUrGrG rUrUrU rUrArC rCrCrA rArGrU rGrGrC rArCrC rGrArG rUrCrG rCrArA rGrArA rArArA rArArC rGrArA rCrC -3'

Supplementary Table 2: Expressed sgRNA sequences

Target	sgRNA	Sequence
Cas9 stoplight	WT	GGACAGUACUCCGCUCGAGUGUUUUAGAGCUAGAAAUAGCAAGUUAAAAUAAGGCU AGUCCGUUAUCAACUUGAAAAAGUGGCACCGAGUCGGUGCUUUUUU
Cas9 stoplight	MS2-2.0	GGACAGUACUCCGCUCGAGUGUUUUAGAGCUAGGCCAACAUGAGGAUCACCCAUGUC UGCAGGGCCUAGCAAGUUAAAAUAAGGCUAGUCCGUUAUCAACUUGGCCAACAUGAG GAUCACCCAUGUCUGCAGGGCCAAGUGGCACCGAGUCGGUGCUUUUUUU
Tet resp. element	WT	GUCUCUAUCACUGAUAGGGAGGUUUUAGAGCUAGAAAUAGCAAGUUAAAAUAAGGC UAGUCCGUUAUCAACUUGAAAAAGUGGCACCGAGUCGGUGCUUUUUU
Tet resp. element	MS2-2.0	GUCUCUAUCACUGAUAGGGAGGUUUUAGAGCUAGGCCAACAUGAGGAUCACCCAUG UCUGCAGGGCCUAGCAAGUUAAAAUAAGGCUAGUCCGUUAUCAACUUGGCCAACAUG AGGAUCACCCAUGUCUGCAGGGCCAAGUGGCACCGAGUCGGUGCUUUUUUU
ABE stoplight	WT	GCUUACUUGUACAGCUCGUCCGUUUUAGAGCUAGAAAUAGCAAGUUAAAAUAAGGC UAGUCCGUUAUCAACUUGAAAAAGUGGCACCGAGUCGGUGCUUUUUU
ABE stoplight	MS2-1.1	GCUUACUUGUACAGCUCGUCCGUUUUAGAGCUAACAUGAGGAUCACCCAUGUUAGCA AGUUAAAAUAAGGCUAGUCCGUUAUCAACUUGGACUUCGGUCCAAGUGGCACCGAG UCGGUGCUUUUUUU
ABE stoplight	MS2-1.2	GCUUACUUGUACAGCUCGUCCGUUUCAGAGCUAAGCACAAGAGUGCAUAGCAAGUUG AAAUAAGGCUAGUCCGUUUACAACUUGGCCAACAUGAGGAUUACCCAUGUCUGCAGG GCCAAGUGGCACCCGAGUCGGGUGCUUUUUUU
ABE stoplight	MS2-2.0	GCUUACUUGUACAGCUCGUCCGUUUUAGAGCUAGGCCAACAUGAGGAUCACCCAUGU CUGCAGGGCCUAGCAAGUUAAAAUAAGGCUAGUCCGUUAUCAACUUGGCCAACAUGA GGAUCACCCAUGUCUGCAGGGCCAAGUGGCACCGAGUCGGUGCUUUUUUU
CCR5 locus	MS2-1.1	GUGACAUCAAUUAUUAUACAUGUUUUAGAGCUAACAUGAGGAUCACCCAUGUUAGC AAGUUAAAAUAAGGCUAGUCCGUUAUCAACUUGGACUUCGGUCCAAGUGGCACCGAG UCGGUGCUUUUUUU

Legend: sgRNA targeting sequence, MS2 aptamer.

Supplementary Table 3: AA sequences of RNA-binding constructs and fluorescently labeled proteins

Construct	Amino acid sequence
MCP-CD63	MASNFTQFVLVDNGGTGDVTVAPSNFANGVAEWISSNSRSQAYKVTCSVRQSSAQNRKYTIKVEVPKGAWRSYLNMELTIPIFATNSDCELIVKAMQGLLKDGNPIPSAIAANSGIYAMASNFTQFVLVDNGGTGDVTVAPSNFANGVAEWISSNSRSQAYKVTCSVRQSSAQNRKYTIKVEVPKGAWRSYLNMELTIPIFATNSDCELIVKAMQGLLKDGNPIPSAIAANSGIYGSSPSTSLYKKAGSEFALKLAVEGGMKCVKFLLYVLLLAFCACAVGLIAVGVGAQLVLSQTIIQGATPGSLLPVVIIAVGVFLFLVAFVGCCGACKENYCLMITFAIFLSLIMLVEVAAAIAGYVFRDKVMSEFNNNFRQQMENYPKNNHTASILDRMQADFKCCGAANYTDWEKIPSMSKNRVPDSCCINVTVGCGINFNEKAIHKEGCVEKIGGWLRKNVLVVAAAALGIAFVEVLGIVFACCLVKSIRSGYEVM*
MCP-PhoCl-CD63	MASNFTQFVLVDNGGTGDVTVAPSNFANGVAEWISSNSRSQAYKVTCSVRQSSAQNRKYTIKVEVPKGAWRSYLNMELTIPIFATNSDCELIVKAMQGLLKDGNPIPSAIAANSGIYAMASNFTQFVLVDNGGTGDVTVAPSNFANGVAEWISSNSRSQAYKVTCSVRQSSAQNRKYTIKVEVPKGAWRSYLNMELTIPIFATNSDCELIVKAMQGLLKDGNPIPSAIAANSGIYGSSYPYDVPDYAVIPDYFKQSFPEGYSWERSMTYEDGGICIATNDITMEGDSFINKIHFKGTNFPPNGPVMQKRTVGWEASTEKMYERDGVLKGDVKMKLLLKGGGHYRCDYRTTYKVKQKPVKLPDYHFVDHRIEILSHDKDYNKVKLYEHAVARNSTDSMDELYKGGSGGMVSKGEETITSVIKPDMKNKLRMEGNVNGHAFVIEGEGSGKPFEGIQTIDLEVKEGAPLPFAYDILTTAFHYGNRVFTKYPRDYKDDDDKLYKKAGSEFALKLAVEGGMKCVKFLLYVLLLAFCACAVGLIAVGVGAQLVLSQTIIQGATPGSLLPVVIIAVGVFLFLVAFVGCCGACKENYCLMITFAIFLSLIMLVEVAAAIAGYVFRDKVMSEFNNNFRQQMENYPKNNHTASILDRMQADFKCCGAANYTDWEKIPSMSKNRVPDSCCINVTVGCGINFNEKAIHKEGCVEKIGGWLRKNVLVVAAAALGIAFVEVLGIVFACCLVKSIRSGYEVM*
MCP-PhoCl-CD9	MASNFTQFVLVDNGGTGDVTVAPSNFANGVAEWISSNSRSQAYKVTCSVRQSSAQNRKYTIKVEVPKGAWRSYLNMELTIPIFATNSDCELIVKAMQGLLKDGNPIPSAIAANSGIYAMASNFTQFVLVDNGGTGDVTVAPSNFANGVAEWISSNSRSQAYKVTCSVRQSSAQNRKYTIKVEVPKGAWRSYLNMELTIPIFATNSDCELIVKAMQGLLKDGNPIPSAIAANSGIYGSSYPYDVPDYAVIPDYFKQSFPEGYSWERSMTYEDGGICIATNDITMEGDSFINKIHFKGTNFPPNGPVMQKRTVGWEASTEKMYERDGVLKGDVKMKLLLKGGGHYRCDYRTTYKVKQKPVKLPDYHFVDHRIEILSHDKDYNKVKLYEHAVARNSTDSMDELYKGGSGGMVSKGEETITSVIKPDMKNKLRMEGNVNGHAFVIEGEGSGKPFEGIQTIDLEVKEGAPLPFAYDILTTAFHYGNRVFTKYPRDYKDDDDKLYKKAGSEFALKLPVKGGTKCIKYLLFGFNFIFWLAGIAVLAIGLWLRFDSQTKSIFEQETNNNNSSFYTGVYILIGAGALMMLVGFLGCCGAVQESQCMLGLFFGFLLVIFAIEIAAAIWGYSHKDEVIKEVQEFYKDTYNKLKTKDEPQRETLKAIHYALNCCGLAGGVEQFISDICPKKDVLETFTVKSCPDAIKEVFDNKFHIIGAVGIGIAVVMIFGMIFSMILCCAIRRNREMV*
MCP-PhoCl-CD81	MASNFTQFVLVDNGGTGDVTVAPSNFANGVAEWISSNSRSQAYKVTCSVRQSSAQNRKYTIKVEVPKGAWRSYLNMELTIPIFATNSDCELIVKAMQGLLKDGNPIPSAIAANSGIYAMASNFTQFVLVDNGGTGDVTVAPSNFANGVAEWISSNSRSQAYKVTCSVRQSSAQNRKYTIKVEVPKGAWRSYLNMELTIPIFATNSDCELIVKAMQGLLKDGNPIPSAIAANSGIYGSSYPYDVPDYAVIPDYFKQSFPEGYSWERSMTYEDGGICIATNDITMEGDSFINKIHFKGTNFPPNGPVMQKRTVGWEASTEKMYERDGVLKGDVKMKLLLKGGGHYRCDYRTTYKVKQKPVKLPDYHFVDHRIEILSHDKDYNKVKLYEHAVARNSTDSMDELYKGGSGGMVSKGEETITSVIKPDMKNKLRMEGNVNGHAFVIEGEGSGKPFEGIQTIDLEVKEGAPLPFAYDILTTAFHYGNRVFTKYPRDYKDDDDKLYKKAGSEFALKLGVEGCTKCIKYLLFVFNFVFWLAGGVILGVALWLRHDPQTTNLLYLELGDKPAPNTFYVGIYILIAVGAVMMFVGFLGCYGAIQESQCLLGTFFTCLVILFACEVAAGIWGFVNKDQIAKDVKQFYDQALQQAVVDDDANNAKAVVKTFHETLDCCGSSTLTALTTSVLKNNLCPSGSNIISNLFKEDCHQKIDDLFSGKLYLIGIAAIVVAVIMIFEMILSMVLCCGIRNSSVY*
MCP-PhoCl-ARRDC1	MASNFTQFVLVDNGGTGDVTVAPSNFANGVAEWISSNSRSQAYKVTCSVRQSSAQNRKYTIKVEVPKGAWRSYLNMELTIPIFATNSDCELIVKAMQGLLKDGNPIPSAIAANSGIYAMASNFTQFVLVDNGGTGDVTVAPSNFANGVAEWISSNSRSQAYKVTCSVRQSSAQNRKYTIKVEVPKGAWRSYLNMELTIPIFATNSDCELIVKAMQGLLKDGNPIPSAIAANSGIYGSSYPYDVPDYAVIPDYFKQSFPEGYSWERSMTYEDGGICIATNDITMEGDSFINKIHFKGTNFPPNGPVMQKRTVGWEASTEKMYERDGVLKGDVKMKLLLKGGGHYRCDYRTTYKVKQKPVKLPDYHFVDHRIEILSHDKDYNKVKLYEHAVARNSTDSMDELYKGGSGGMVSKGEETITSVIKPDMKNKLRMEGNVNGHAFVIEGEGSGKPFEGIQTIDLEVKEGAPLPFAYDILTTAFHYGNRVFTKYPRDYKDD

	DDKLYKKAGSEFALKLMGRVQLFEISLSHGRVVYSPGEPLAGTVRVRLGAPLPFRAIRVTCIGSCGVSNKANDTAWVVEEGYFNSSLSLADKGSLPAGEHSFPFQFLLPATAPTSFEGPFGKIVHQVRAAIHTPRFSKDHKCSLVFYILSPLNLNSIPDIEQPNVASATKKFSYKLVKTGSVVLTASTDLRGYVVGQALQLHADVENQSGKDTSPVVASLLQKVSYKAKRWIHDVRTIAEVEGAGVKAWRRAQWHEQILVPALPQSALPGCSLIHIDYYLQVSLKAPEATVTLPVFIGNIAVNHAPVSPRPGLGLPPGAPPLVVPSAPPQEEAEAEAAAGGPHFLDPVFLSTKSHSQRQPLLATLSSVPGAPEPCPQDGSPASHPLHPPLCISTGATVPYFAEGSGGPVPTTSTLILPPEYSSWGYPYEAPPSYEQSCGGVEPSLTPES*
Myristoyl-PhoCl-MCP	MGSSKSKPKDPSQRGGGGSSSSYPYDVPDYAVIPDYFKQSFPEGYSWERSMTYEDGGICIATNDITMEGDSFINKIHFKGTNFPPNGPVMQKRTVGWEASTEKMYERDGVLKGDVKMKLLLKGGGHYRCDYRTTYKVKQKPVKLPDYHFVDHRIEILSHDKDYNKVKLYEHAVARNSTDSMDELYKGGSGGMVSKGEETITSVIKPDMKNKLRMEGNVNGHAFVIEGEGSGKPFEGIQTIDLEVKEGAPLPFAYDILTTAFHYGNRVFTKYPRDYKDDDDKLYSQRGGGGSASNFTQFVLVDNGGTGDVTVAPSNFANGVAEWISSNSRSQAYKVTCSVRQSSAQNRKYTIKVEVPKGAWRSYLNMELTIPIFATNSDCELIVKAMQGLLKDGNPIPSAIAANSGIYAMASNFTQFVLVDNGGTGDVTVAPSNFANGVAEWISSNSRSQAYKVTCSVRQSSAQNRKYTIKVEVPKGAWRSYLNMELTIPIFATNSDCELIVKAMQGLLKDGNPIPSAIAANSGIY*
Free-MCP	MASNFTQFVLVDNGGTGDVTVAPSNFANGVAEWISSNSRSQAYKVTCSVRQSSAQNRKYTIKVEVPKGAWRSYLNMELTIPIFATNSDCELIVKAMQGLLKDGNPIPSAIAANSGIYAMASNFTQFVLVDNGGTGDVTVAPSNFANGVAEWISSNSRSQAYKVTCSVRQSSAQNRKYTIKVEVPKGAWRSYLNMELTIPIFATNSDCELIVKAMQGLLKDGNPIPSAIAANSGIYGSS*
H2B-mTag-BFP2	MPEPAKSAPAPKKGSKKAVTKAQKKGGKKRKRSRKESYSIYVYKVLKQVHPDTGISSKAMGIMNSFVNDIFERIAGEASRLAHYNKRSTITSREIQTAVRLLLPGELAKHAVSEGTKAITKYTSAKGGGGSAAAMVSKGEELIKENMHMKLYMEGTVDNHHFKCTSEGEGKPYEGTQTMRIKVVEGGPLPFAFDILATSFLYGSKTFINHTQGIPDFFKQSFPEGFTWERVTTYEDGGVLTATQDTSLQDGCLIYNVKIRGVNFTSNGPVMQKKTLGWEAFTETLYPADGGLEGRNDMALKLVGGSHLIANAKTTYRSKKPAKNLKMPGVYYVDYRLERIKEANNETYVEQHEVAVARYCDLPSKLGHKLN*
Cas9-mStayGold-P2A-BlastR	M**[Cas9]**KRPAATKKAGQAKKKKDYKDDDDKGSGGGGSMASTPFKFQLKGTINGKSFTVEGEGEGNSHEGSHKGKYVCTSGKLPMSWAALGTSFGYGMKYYTKYPSGLKNWFHEVMPEGFTYDRHIQYKGDGSIHAKHQHFMKNGTYHNIVEFTGQDFKENSPVLTGDMNVSLPNDVQHIPRDDGVECPVTLLYPLLSDKSKCVEAHQNTICKPLHNQPAPDVPYHWIRKQYTQSKDDTEERDHICQSETLEAHLHHHHHHGSGATNFSLLKQAGDVEENPGPMAKPLSQEESTLIERATATINSIPISEDYSVASAALSSDGRIFTGVNVYHFTGGPCAELVVLGTAAAAAAGNLTCIVAIGNENRGILSPCGRCRQVLLDLHPGIKAIVKDSDGQPTAVGIRELLPSGYVWEG*
ABE8e-mStayGold	MKRTADGSEFESPKKKRKV**[ABE8e]**SGGSKRTADGSEFGGGGSMASTPFKFQLKGTINGKSFTVEGEGEGNSHEGSHKGKYVCTSGKLPMSWAALGTSFGYGMKYYTKYPSGLKNWFHEVMPEGFTYDRHIQYKGDGSIHAKHQHFMKNGTYHNIVEFTGQDFKENSPVLTGDMNVSLPNDVQHIPRDDGVECPVTLLYPLLSDKSKCVEAHQNTICKPLHNQPAPDVPYHWIRKQYTQSKDDTEERDHICQSETLEAHLSGGSKRTADGSEFEPKKKRKV*

Legend: MCP, Linker, Photocleavable domain (PhoCl), EV-targeting moiety, NLS, Blasticidin resistance, P2A domain, HA-tag, FLAG-tag, His-tag, Fluorescent protein, **[Cas9 / ABE8e protein]**.

Supplementary Table 4: DNA plasmid overview

Name	Function	Source
pMD2.G	VSV-G expression	Addgene #12259
PSPAX2	2nd generation lentiviral packaging plasmid	Addgene #12660
lentiCas9-Blast	Transient/stable expression of spCas9, labeled with a Flag-tag	Addgene #52962
pCMV-ABE7.10	Transient expression of adenine base editor ABE7.10	Addgene #102919
pCMV-ABE8e	Transient expression of adenine base editor ABE8e	Addgene #138489
pCMV-Abe8e-dimer	Transient expression of adenine base editor ABE8e-dimer, containing an additional ecTadA domain	Addgene #138490
Sp-dCas9-VPR	Transient expression of transcriptional activatory dCas9-VPR	Addgene #63798
lentiGuide-puro	Transient/stable expression of WT sgRNAs	Addgene #52963
lentiGuide-stoplight-puro	Transient/stable expression of WT sgRNA targeting the stoplight reporter construct	Previously published[4]
lentiGuide-TRE-puro	Transient/stable expression of WT sgRNA targeting the Tet Responsive Element	Previously published[4]
Lentiguide-ABE stoplight-puro	Transient/stable expression of WT sgRNA targeting the adenine base editor stoplight reporter construct	Generated for this study
lenti-sgRNA(MS2)-zeo	Transient/stable expression of MS2-2.0 sgRNAs	Addgene #61427
lenti-Stoplight-sgRNA(MS2)-zeo	Transient/stable expression of a MS2-2.0 sgRNA targeting the stoplight reporter construct	Generated for this study
lenti-TRE-sgRNA(MS2)-zeo	Transient/stable expression of a MS2-2.0 sgRNA targeting the Tet Responsive Element	Generated for this study
lenti-ABE Stoplight-sgRNA(MS2)-zeo	Transient/stable expression of a MS2-2.0 sgRNA targeting the adenine base editor stoplight reporter construct	Generated for this study
lenti-gRNA puro	Transient/stable expression of sgRNAs or crRNAs, no gRNA backbone present in backbone	Addgene #84752
lenti-ABE Stoplight 1.1-gRNA puro	Transient/stable expression of a MS2-1.1 sgRNA targeting the adenine base editor stoplight reporter construct	Generated for this study
lenti-ABE Stoplight 1.2-gRNA puro	Transient/stable expression of a MS2-1.2 sgRNA targeting the adenine base editor stoplight reporter construct	Generated for this study
pInducer20	2nd generation lentiviral transfer plasmid for doxycycline-inducible protein expression	Addgene #44012
pDONR221-eGFP	Used to clone eGFP into pInducer20 using the Gateway LR Clonase II Enzyme Mix	Addgene #25899
pInducer20-eGFP	Transient/stable expression of doxycycline / dCas9-VPR-inducible eGFP expression	Generated for this study
pHAGE2-EF1a-IRES-PuroR	2nd generation lentiviral transfer plasmid for protein expression	Darrel Kotton Lab
pHAGE2-EF1a-IRES-NeoR	2nd generation lentiviral transfer plasmid for protein expression	Darrel Kotton Lab
pHAGE2-CMV-Stoplight-IRES-NeoR	Transient/stable expression expression of the Cas9 stoplight reporter construct	Previously published[4]
pHAGE2-EF1a-ABE Stoplight-IRES-NeoR	Transient/stable expression of the adenine base editor / HDR stoplight reporter construct	Previously published[5]

pHAGE2-EF1a- MCP-CD63-IRES-PuroR	Transient/stable expression of tandem MCPs fused to CD63	Generated for this study
pHAGE2-EF1a- MCP-PhoCl-CD63-IRES-PuroR	Transient/stable expression of tandem MCPs fused to CD63 with a photocleavable domain	Generated for this study
pHAGE2-EF1a- MCP-PhoCl-CD9-IRES-PuroR	Transient/stable expression of tandem MCPs fused to CD9 with a photocleavable domain	Generated for this study
pHAGE2-EF1a- MCP-PhoCl-CD81-IRES-PuroR	Transient/stable expression of tandem MCPs fused to CD81 with a photocleavable domain	Generated for this study
pHAGE2-EF1a- MCP-PhoCl-ARRDC1-IRES-PuroR	Transient/stable expression of tandem MCPs fused to ARRDC1 with a photocleavable domain	Generated for this study
pHAGE2-EF1a- myristoyl-PhoCl-MCP-IRES-PuroR	Transient/stable expression of a myristoylation tag fused to tandem MCPs with a photocleavable domain	Generated for this study
pHAGE2-EF1a-Free MCP-IRES-PuroR	Transient/stable expression of cytosolic tandem MCPs	Generated for this study
FUW-EF1a-H2B-mTAG_BFP-IRES-PuroR	Transient/stable expression of mTag-BFP2-labeled Histone 2B for nuclear localization	Generated for this study
pLentiCas9-mStayGold-E138D	Transient/stable expression of mStayGold-labeled spCas9	Generated for this study
pCMV Abe8e-mStayGold	Transient/stable expression of mStayGold-labeled ABE8e	Generated for this study

Supplementary Table 5: PCR and next generation sequencing primers

Target	Orientation	Application	Sequence
MS2 sgRNA	Forward	ddPCR/qPCR	5'-GACAGTACTCCGCTCGAGTG-3'
Spike-in	Forward	ddPCR/qPCR	5'-CCAGATTACTTCCATTTC CGCC-3'
MS2 sgRNA & Spike-in	Reverse	ddPCR/qPCR	5'-CGACTCGGTGCCACTTGG-3'
CCR5 locus	Forward	T7E1/BsaHI	5’-CAACAGAGCCAAGCTCTCCAT-3’
CCR5 locus	Reverse	T7E1/BsaHI	5’-CCTGGGAGAGACGCAAACAC-3’
Cas9 stoplight (SL) target seq	Forward	NGS/T7E1	5’-GAAGGGCGAGATCAAGCAGA-3’
Cas9 stoplight (SL) target seq	Reverse	NGS/T7E1	5’-GGTCTTGTAGTTGCCGTCGT-3’
chr2:+120580702 (SL off-target 1)	Forward	NGS	5’-CTCTTGCTGACTCGACTGGT-3’
chr2:+120580702 (SL off-target 1)	Reverse	NGS	5’-GAGATAACACCCGGGCCTG-3’
chr4:157825214 (SL off-target 2)	Forward	NGS	5’-GTGCAGGGAAAACACCATAGG-3
chr4:157825214 (SL off-target 2)	Reverse	NGS	5’-CAGGTGGAGGTGGTGAGC-3’
chr14:104942109 (SL off-target 3)	Forward	NGS	5’-GGCATCTTGAACTTGGGCAT-3’
chr14:104942109 (SL off-target 3)	Reverse	NGS	5’-GCCCAGTTTCAAGGTACCCA-3’
chr5:180630776 (SL off-target 4)	Forward	NGS	5’-CTCGCAGTCTCGCACCAC-3’
chr5:180630776 (SL off-target 4)	Reverse	NGS	5’-CTGTAGTGTGCGCCCAAAC-3’
chr8:135581932 (SL off-target 5)	Forward	NGS	5’-TGACTCTCTTGGTTACAGGGG-3’
chr8:135581932 (SL off-target 5)	Reverse	NGS	5’-AGTCAATCTGATGAACAAGGCTG-3’

Supplementary Information References

1. Kanda, T., Sullivan, K. F. & Wahl, G. M. Histone-GFP fusion protein enables sensitive analysis of chromosome dynamics in living mammalian cells. *Curr. Biol.* **8**, 377–385 (1998).

2. Konermann, S. *et al.* Genome-scale transcriptional activation by an engineered CRISPR-Cas9 complex. *Nature* **517**, 583–588 (2015).

3. Hsu, P. D. *et al.* DNA targeting specificity of RNA-guided Cas9 nucleases. *Nat. Biotechnol. 2013 319* **31**, 827–832 (2013).

4. de Jong, O. G. *et al.* A CRISPR-Cas9-based reporter system for single-cell detection of extracellular vesicle-mediated functional transfer of RNA. *Nat. Commun.* **11**, (2020).

5. Öktem, M., Mastrobattista, E. & de Jong, O. G. Amphipathic Cell-Penetrating Peptide-Aided Delivery of Cas9 RNP for In Vitro Gene Editing and Correction. *Pharmaceutics* **15**, (2023).
