# Supplementary material for: A modular strategy for extracellular vesicle-mediated CRISPR-Cas9 delivery through aptamer-based loading and UV-activated cargo release
Source: Nat Commun. 2025 Nov 21;16:10309. doi: 10.1038/s41467-025-65995-3 (PMC12639036; doi:10.1038/s41467-025-65995-3)
Supplement: Supplementary file 2 — Reporting Summary [file 41467_2025_65995_MOESM2_ESM.pdf]

nature portfolio

Corresponding author(s): Olivier Gerrit de Jong

Last updated by author(s): September 22, 2025

Reporting Summary

Nature Portfolio wishes to improve the reproducibility of the work that we publish. This form provides structure for consistency and transparency in reporting. For further information on Nature Portfolio policies, see our Editorial Policies and the Editorial Policy Checklist.

Statistics

For all statistical analyses, confirm that the following items are present in the figure legend, table legend, main text, or Methods section.

n/a	Confirmed	
☐	☒	The exact sample size (*n*) for each experimental group/condition, given as a discrete number and unit of measurement
☒	☐	A statement on whether measurements were taken from distinct samples or whether the same sample was measured repeatedly
☐	☒	The statistical test(s) used AND whether they are one- or two-sided<br>*Only common tests should be described solely by name; describe more complex techniques in the Methods section.*
☒	☐	A description of all covariates tested
☐	☒	A description of any assumptions or corrections, such as tests of normality and adjustment for multiple comparisons
☐	☒	A full description of the statistical parameters including central tendency (e.g. means) or other basic estimates (e.g. regression coefficient) AND variation (e.g. standard deviation) or associated estimates of uncertainty (e.g. confidence intervals)
☐	☒	For null hypothesis testing, the test statistic (e.g. *F*, *t*, *r*) with confidence intervals, effect sizes, degrees of freedom and *P* value noted<br>*Give P values as exact values whenever suitable.*
☒	☐	For Bayesian analysis, information on the choice of priors and Markov chain Monte Carlo settings
☒	☐	For hierarchical and complex designs, identification of the appropriate level for tests and full reporting of outcomes
☒	☐	Estimates of effect sizes (e.g. Cohen's *d*, Pearson's *r*), indicating how they were calculated

Our web collection on statistics for biologists contains articles on many of the points above.

Software and code

Policy information about availability of computer code

Data collection	Data was collected using up-to-date software provided by the manufacturer for all the equipment listed in the Methods section.
Data analysis	Fluorescence microscopy were processed using ImageJ v1.54d, flow cytometry data was analyzed using FlowJo v10, statistical analyses were performed using Graphpad Prism 10.0.2. Nanoparticle tracking analysis measurements were analyzed using NTA software v3.4. ddPCR analysis was performed using QuantaSoft (v. 1.7.4.0917). Confocal images were analyzed for protein expression and co-localization using Perkin-Elmer Columbus Image Data Storage and Analysis System v2.7.1. Next generation sequencing was analyzed using the online Cas-Analyzer software package.

For manuscripts utilizing custom algorithms or software that are central to the research but not yet described in published literature, software must be made available to editors and reviewers. We strongly encourage code deposition in a community repository (e.g. GitHub). See the Nature Portfolio guidelines for submitting code & software for further information.

Data

Policy information about availability of data

All manuscripts must include a data availability statement. This statement should provide the following information, where applicable:

- Accession codes, unique identifiers, or web links for publicly available datasets
- A description of any restrictions on data availability
- For clinical datasets or third party data, please ensure that the statement adheres to our policy

The datasets generated during and/or analysed during the current study are available from the corresponding author on request.

Research involving human participants, their data, or biological material

Policy information about studies with human participants or human data. See also policy information about sex, gender (identity/presentation), and sexual orientation and race, ethnicity and racism.

Reporting on sex and gender	No sex- or gender-based data were collected and/or analyzed in this study.
Reporting on race, ethnicity, or other socially relevant groupings	No data on race, ethnicity, or any other socially relevant groupings were collected and/or analyzed in this study.
Population characteristics	No data on population characteristics were collected and/or analyzed in this study.
Recruitment	No human participants were included and/or analyzed in this study.
Ethics oversight	All work performed with biological materials (cell lines, lentiviral expression systems, transfections) were performed in accordance with regulations of the Dutch National Institute for Public Health and Environment, under Biosafety Permit IG 97-236, under oversight of the Utrecht University Biosafety Office.

Note that full information on the approval of the study protocol must also be provided in the manuscript.

Field-specific reporting

Please select the one below that is the best fit for your research. If you are not sure, read the appropriate sections before making your selection.

☒ Life sciences ☐ Behavioural & social sciences ☐ Ecological, evolutionary & environmental sciences

For a reference copy of the document with all sections, see nature.com/documents/nr-reporting-summary-flat.pdf

Life sciences study design

All studies must disclose on these points even when the disclosure is negative.

Sample size	Experiments were performed 3-5 times, and analyzed using the statistical analyses listed in the manuscript. No sample size-calculation was performed prior to experiments. Sample sizes were based on prior experiences and dose response curves (Fig. S8B, S10A, S10C).
Data exclusions	No data points were excluded in the statistical analysis presented in this manuscript.
Replication	Experiments were multiple times, as described in the "Sample Size" section, to confirm reproducibility.
Randomization	Samples were not randomized during this study. As all samples were measured at the same time using the same machine settings and parameters in software-mediated analysis, it is unlikely that this affected the experimental results and/or the outcomes of this study.
Blinding	Samples were not blinded throughout this study. As mentioned above, we do not expect that this affects the outcome of this study as analyses were not done manually, but through software-mediated analysis.

Reporting for specific materials, systems and methods

We require information from authors about some types of materials, experimental systems and methods used in many studies. Here, indicate whether each material, system or method listed is relevant to your study. If you are not sure if a list item applies to your research, read the appropriate section before selecting a response.

Materials & experimental systems

n/a	Involved in the study
☐	☒ Antibodies
☐	☒ Eukaryotic cell lines
☒	☐ Palaeontology and archaeology
☒	☐ Animals and other organisms
☒	☐ Clinical data
☒	☐ Dual use research of concern
☒	☐ Plants

Methods

n/a	Involved in the study
☒	☐ ChIP-seq
☐	☒ Flow cytometry
☒	☐ MRI-based neuroimaging

Antibodies

Antibodies used	As described in the methods section:<br>The following primary antibodies were used: ALIX 1:1000 (Thermo Fisher Scientific, MA1-83977), Calnexin 1:1000 (GeneTex, GTX101676), CD63 1:1000 (AB8219), Syntenin 1:500 (Origene, OTI2H6), TSG101 1:1000 (Abcam, ab30871), and H2B 1:1000 (Abcam, ab52599), GAPDH 1:500 (Abcam, AB9485) or α-Flag 1:1000 (Sigma-Aldrich, F1804), and the following secondary antibodies were used: anti-rabbit IgG conjugated to AlexaFluor 680 (Thermo Fisher Scientific, A-21076), anti-mouse IgG conjugated to AlexaFluor 680 (Thermo Fisher Scientic, A-21057), anti-rabbit IgG conjugated to IRDye 800CW (926-32211 , LI-COR Biosciences), or anti-mouse IgG conjugated to IRDye 800CW (926-32212, LI-COR Biosciences). Total protein stain was performed using No-Stain Protein Labeling Reagent (ThermoFisher Scientific).
Validation	Alix MA1-83977 was verified by Sigma-Aldrich by probing Alix WT and knockdown samples.<br>Calnexin GTX101676 was verified by GeneTex by probing Calnexin WT and knockdown samples.<br>CD9 ab92726 was verified by Abcam by probing CD9 WT and KO samples.<br>CD63 AB8219 was not verified using KD/KO by Abcam, but Abcam lists its use in 63 publications, shows a band at the expected height, and shows enrichment in EVs as compared to cell lysate, as is expected for CD63<br>H2B AB52599 was not verified using KD/KO by Abcam, but Abcam lists its use in 17 publications, shows a band at the expected height, and shows a negative enrichment in EVs as compared to cell lysate, as is expected for H2B<br>α-Flag F1804 is a commonly used, well characterized antibody. Searching peer reviewed papers via Sigma reveals 8972 manuscripts listing F1804.<br>Syntenin OTI2H6 was verified by Origene by probing various cell lines and tissue samples, as well as HEK293T syntenin overexpression samples

Eukaryotic cell lines

Policy information about cell lines and Sex and Gender in Research

Cell line source(s)	As listed in the manuscript: HEK293T cells (CRL-3216), MCF-7 cells (HTB-22), and MDA-MB-231 cells (HTB-26) were obtained from the American Type Culture Collection (ATCC). T47D cells (85102201) were obtained from Sigma-Aldrich.
Authentication	After receiving the cells from the ATCC / Sigma-Aldrich, cell lines were authenticated by morphological assessment through light microscopy.
Mycoplasma contamination	All cell lines were regularly tested for mycoplasma throughout the project, and tested negative for mycoplasma.
Commonly misidentified lines<br>(See ICLAC register)	No commonly misidentified lines were used in this study.

Plants

Seed stocks	No plants were used in this study.
Novel plant genotypes	No plants were used in this study.
Authentication	No plants were used in this study.

April 2023

Flow Cytometry

Plots

Confirm that:

☒ The axis labels state the marker and fluorochrome used (e.g. CD4-FITC).

☒ The axis scales are clearly visible. Include numbers along axes only for bottom left plot of group (a 'group' is an analysis of identical markers).

☒ All plots are contour plots with outliers or pseudocolor plots.

☒ A numerical value for number of cells or percentage (with statistics) is provided.

Methodology

Sample preparation	As listed in the methods section "After fluorescence microscopy, cells were gently washed with 0.5 ml PBS and trypsinized for 5 minutes using 250 µl TrypLE Express (Thermo Fisher Scientific). Cells were then transferred to 1.5 ml tubes using a similar volume of cell culture medium containing 10% FBS. Cells were centrifuged for 5 minutes at 300 x g, washed in 1 ml 1% FBS in PBS, and centrifuged for 5 minutes at 300 x g. After supernatant removal, cells were resuspended in 200 µl 1% FBS in PBS, transferred to 96-well U-bottom Falcon plates (Fisher Scientific), and analyzed by flow cytometry. "
Instrument	As listed in the methods section cells were "(...) analyzed on a BD FACSCanto II flow cytometry system (BD Biosciences), a CytoFLEX LX Flow Cytometer (Beckmann Coulter), or a BD LSRFortessa Cell Analyzer (BD Biosciences)."
Software	Flow cytometry results were analyzed using FlowJo v10 software.
Cell population abundance	For flow cytometry, at least 10.000 events/sample (and generally > 25.000 events/sample) were analyzed to ensure sufficient resolution and accuracy for measurement and analysis.
Gating strategy	Gating strategies for all reporter cell lines are shown in Supplementary Figure 1.<br><br>For Cas9 Frameshift Stoplight reporter cells and for Cas9 Base editor / HDR reporter cells:<br>- Firstly, cell debris was gated out and cells were selected using FSC and SSC.<br>- Then, single cells were selected by plotting SSC-A vs SSC-H.<br>- Next, cell positive for the reporter construct (generally > 95%) were selected the mCherry signal. For the FACSCanto II flow cytometry system this was done using the 633 nm laser (FSC-APC plot), for the LSRFortessa this was done using the Yellow-Green laser (FSC-YeGr plot).<br>- Lastly, for the reporter cells (mCherry+ gated), reporter activation was assessed by measuring eGFP expression using a blue (488 nm) laser (FSC-Blue plot).<br><br>For transcriptional activation reporter cells (dCas9-VPR):<br>- Firstly, cell debris was gated out and cells were selected using FSC and SSC.<br>- Then, single cells were selected by plotting SSC-A vs SSC-H.<br>- Lastly, the eGFP mean fluorescence intensity was measured using a blue (488 nm) laser (Histogram plot).

☒ Tick this box to confirm that a figure exemplifying the gating strategy is provided in the Supplementary Information.
